# ENO1 Is More Abundant than ENO2 on the Surface of Metastatic and Neuroendocrine Prostate Cancer Cells and Upregulated in High-Risk Prostate Tumors

**DOI:** 10.3390/ijms27219714

**Published:** 2026-10-29

**Authors:** Krystal R. Santiago, Alfonso M. Durán, Lemti M. Nyirendah, Julia Soliz, Ann Morcos, Adelaide V. Makamure, Nathaly P. Manrique, Kathleen Escobar-Acuña, Kristen Whitley, Shriya Roy, Nathan R. Wall, Bhaskar Das, Frankis G. Almaguel, Carlos A. Casiano

**Affiliations:** 1 Center for Health Disparities and Molecular Medicine, Loma Linda University School of Medicine, Mortensen Hall 142, 11085 Campus Street, Loma Linda, CA 92350, USA; krystalsantiago@students.llu.edu (K.R.S.); aduran@llu.edu (A.M.D.); lnyirendah@llu.edu (L.M.N.); jsoliz@students.llu.edu (J.S.); amakamure@students.llu.edu (A.V.M.); nmanrique@students.llu.edu (N.P.M.); kescobaracuna@students.llu.edu (K.E.-A.); kristen.whitley140@gmail.com (K.W.); shriyaroy2007@gmail.com (S.R.); falmaguel@llu.edu (F.G.A.); 2 Department of Basic Sciences, Loma Linda University School of Medicine, Loma Linda, CA 92350, USA; 3 Department of Pathology and Human Anatomy, Loma Linda University School of Medicine, Loma Linda, CA 92350, USA; amorcos@llu.edu (A.M.); nwall@llu.edu (N.R.W.); 4 Department of Radiation Medicine, James M. Slater, MD Proton Treatment and Research Center, Loma Linda University Health, Loma Linda, CA 92350, USA; 5 Department of Pharmaceutical Sciences, School of Pharmacy and Pharmaceutical Sciences, University at Buffalo, Buffalo, NY 14215, USA; bhaskard@buffalo.edu; 6 Loma Linda University Cancer Center, Loma Linda, CA 92354, USA

**Keywords:** antibody targeting, ENO1, ENO2, gene silencing, Gleason score, mCRPC, NEPC, PSMA, surface localization, tumor expression, theranostics

## Abstract

Metastatic castration-resistant prostate cancer (mCRPC) remains lethal, and lineage plasticity drives a subset of tumors to neuroendocrine prostate cancer (NEPC), most often treatment-emergent (t-NEPC). Prostate-specific membrane antigen (PSMA), the target of radioligand therapies for advanced prostate cancer (PCa), is heterogeneously expressed across metastatic sites but lost during neuroendocrine differentiation, excluding many patients from these therapies. The glycolytic enzyme Enolase 1 (ENO1) is a promising alternative target because it localizes to the surface of human tumors and promotes cancer growth and metastasis. However, it remains understudied in advanced PCa compared with ENO2 (neuron-specific enolase), a canonical neuroendocrine marker. We report here that total and cell surface ENO1 are more abundant than ENO2 in PSMA-negative mCRPC and NEPC cells. ENO1 silencing reduced cell proliferation and clonogenic growth even when triggering ENO2 upregulation. Targeting cell surface ENO1 with a monoclonal antibody decreased proliferation, clonogenicity, and tumorsphere formation in mCRPC cells, including docetaxel-resistant cells. This was associated with S-phase accumulation, elevated phospho histone p-H2A.X, and increased apoptosis. Significantly higher transcript expression of *ENO1* than *ENO2* was observed in clinical prostate tumors, including high-Gleason and neuroendocrine tumors, concomitant with elevated expression of their transcriptional regulator MYC. These findings position cell-surface ENO1 as a therapeutic and theranostic target for PSMA-low, treatment-refractory PCa.

## 1. Introduction

Prostate cancer (PCa) is the most diagnosed malignancy and the second leading cause of cancer-related death among men in the United States [1]. Low-risk PCa is often managed with active surveillance, whereas high-risk localized disease is treated with radical prostatectomy or radiation therapy, often combined with androgen deprivation therapy (ADT) [2,3,4]. Despite curative-intent treatment, approximately one-third of patients develop biochemical recurrence [2,3,4]. As the disease progresses, treatment is guided by metastatic burden and hormonal sensitivity. Metastatic disease is typically treated with a combination of ADT and androgen receptor pathway inhibitors (ARPI). In metastatic castration-resistant prostate cancer (mCRPC), chemotherapy with taxane drugs such as docetaxel (DTX) and cabazitaxel is used [5,6,7,8,9]. However, resistance to ARPI and taxanes, and cross-resistance between these drugs, contribute to disease progression and patient mortality in mCRPC [10,11,12].

Prostate-specific membrane antigen (PSMA)-targeted radioligand therapy (PSMA-RLT), such as the Food and Drug Administration (FDA)-approved agent Pluvicto^®^ (lutetium-177-PSMA-617) has demonstrated clinical benefits in patients with PSMA-positive mCRPC despite progression of current standard of care therapeutic interventions (ADT, ARPI and taxanes [13]. The phase III PSMAfore trial demonstrated that ^177^Lu-PSMA-617 significantly prolonged radiographic progression-free survival compared with a change in ARPI therapy in taxane-naïve patients with progressive PSMA-positive mCRPC [14]. However, a substantial subset of mCRPC, including neuroendocrine prostate cancer (NEPC) and other androgen receptor (AR) low or negative tumors, exhibits low or heterogeneous PSMA expression, limiting the effectiveness of PSMA-targeted imaging and therapies [15,16,17]. Treatment-emergent NEPC (t-NEPC) commonly develops as a mechanism of resistance to treatment and is characterized by loss of AR signaling and PSMA expression, and gain of neuroendocrine markers such as chromogranin A (CHGA), neuron-specific enolase (NSE, ENO2), somatostatin receptors (SSTRs), and synaptophysin (SYP), together with inactivation of key tumor suppressor genes (e.g., *TP53* and *PTEN*) and activation of oncogenic drivers (e.g., *MYCN*) [18,19,20,21,22]. Collectively, these alterations promote genomic instability, therapy resistance, and lineage plasticity [20,21,22]. Therefore, identifying alternative, targetable cell surface proteins that are stably expressed in PSMA-low/negative mCRPC and NEPC tumor subtypes remains critical for expanding targeted therapy options [13,15,23].

Glycolytic enzymes, particularly Enolase-1 (ENO1, α-enolase) and Enolase-2 (ENO2, γ-enolase, NSE), have emerged as potential cancer-associated targets due to their high tumor expression and ability to localize to the cancer cell surface [24,25,26,27]. Their targeting may broaden therapeutic avenues in mCRPC, particularly in NEPC. ENO1 is a multifunctional glycolytic enzyme with ‘moonlighting’ functions that is considered a key cancer-related protein [24,25,26]. Its isoform ENO2 is predominantly expressed in neurons and neuroendocrine tissues [27,28]. Cancer cells often exhibit increased glycolysis, despite adequate oxygen availability, a metabolic phenomenon known as the Warburg effect [29]. A key regulator of this metabolic reprogramming is Myc-binding protein 1 (MBP-1), an alternative translation product of the *ENO1* gene [30]. Under low-glucose conditions, MBP-1 is upregulated and translocates to the nucleus, where it represses *MYC* (encoding c-Myc), resulting in decreased ENO1 transcription, glycolytic activity, and cell proliferation [30,31]. Conversely, high glucose conditions reduce MBP-1 expression, leading to c-Myc-mediated ENO1 upregulation and supporting glycolysis and proliferation [30,31,32]. 

ENO1 is frequently overexpressed in cancer cells, where it supports glycolysis and promotes signaling programs associated with proliferation, invasion, migration, metastasis, chemoresistance, and epithelial–mesenchymal transition (EMT) [33,34,35,36,37,38,39]. In addition to its intracellular metabolic function, ENO1 can localize to the tumor cell surface, where it acts as a plasminogen receptor, facilitating plasmin generation, extracellular matrix (ECM) degradation and cell invasion and metastasis [39,40,41]. Furthermore, cell surface or extracellular ENO1 enhances glycolytic activity by influencing transcriptional programs of glycolytic enzymes and transporters, and through interactions with cell surface proteins associated with glycolysis regulation [39,42,43,44]. ENO1-mediated metabolic adaptation and therapy resistance have been reported in pancreatic, lung, glioblastoma, colorectal, gastric, breast, and head and neck cancers [35,37,38,40,45,46,47,48,49,50,51,52,53,54,55,56]. However, our knowledge of these mechanisms in PCa, particularly in NEPC, remains limited. 

Given the extracellular accessibility of ENO1 and its role in cancer-associated metabolism and aggressive behavior, and the established association of ENO2 with neuroendocrine differentiation, both enzymes warrant investigation as candidate targets for next-generation radioligand and metabolism-directed therapeutic development, as well as radiosensitizing agents in PSMA-low/negative mCRPC, including NEPC. In this study, we characterized the expression and surface localization of ENO1 and ENO2 in mCRPC and NEPC cells to evaluate their potential as targetable antigens to reduce prostate tumor cell survival and proliferation. We also correlated their transcript expression levels with Gleason scores and neuroendocrine features in clinical prostate tumors.

## 2. Results

### 2.1. Expression of ENO1 and ENO2 in DTX-Sensitive and -Resistant mCRPC Cells

The PCa cell lines PC3 (derived from bone metastasis) and DU145 (derived from brain metastasis), along with their DTX-resistant variants, are widely used as pre-clinical models of aggressive, poorly differentiated mCRPC, characterized by loss of AR and prostate specific antigen (PSA). These models also display features of neuroendocrine differentiation, including increased CHGA and ENO2 (NSE) expression relative to AR-positive adenocarcinoma lines [57,58,59], as well as reduced PSMA expression (Supplementary Figure S1A,B), consistent with a therapy-resistant, lineage-plastic mCRPC state that shares features with t-NEPC. Although CD56 (also known as neural cell adhesion molecule 1, NCAM1) has been reported as a neuroendocrine-associated marker [60], it was not detected in our DTX-sensitive and -resistant PC3 and DU145 cells (Supplementary Figure S1C). Thus, these cells should not be considered as established t-NEPC models. The non-NEPC PCa cell line MDA-PCa-2b (AR+), derived from a bone metastasis of an African American patient with androgen-independent adenocarcinoma, expressed high levels of PSMA with little CHGA and ENO2 expression (Supplementary Figure S1A,B). The lymph node metastasis-derived cell line LNCaP (ARhi/GRlow) and its enzalutamide-resistant variant LNCaP-ENZR (ARlow/GRhi) exhibited CHGA expression with varying expression levels of PSMA (Supplementary Figure S1A). LNCaP cells, which are generally considered non-NEPC [57], also showed minimal ENO2 expression; however, they exhibit marked lineage plasticity and after chronic treatment with the ARPI drug enzalutamide, can acquire resistance with substantial increase in ENO2 expression (Supplementary Figure S1B). This is consistent with the observation that LNCaP cells undergoing AR signaling inhibition develop features of NEPC [61]. The patient-derived xenograft 22RV1 cell line (AR+/GRlow) and its DTX-resistant counterpart 22RV1-DR expressed CHGA and ENO2, with minimal PSMA expression (Supplementary Figure S1A,B). Likewise, the well-established NEPC cell lines NCI-H660 and LASCPC-01 [22,62,63] expressed CHGA, ENO2, and CD56 with no PSMA expression (Supplementary Figure S1A–C). These results show substantial heterogeneity in the expression of PSMA and NEPC-related markers across a broad panel of PCa cell models.

The neuroendocrine markers and phenotype in some of these cell lines reflect an adaptation process in which prostate adenocarcinoma cells evolve in response to therapy-induced stress, driving t-NEPC, a highly malignant variant associated with therapeutic resistance and poor clinical outcomes [18]. Based on these features and prior reports, in this study we refer to PC3, DU145, and their DTX-resistant derivatives (PC3-DR, DU145-DR) as mCRPC models that share molecular and phenotypic characteristics with t-NEPC, and to NCI-H660 and LASCPC-01 as bona fide NEPC models. NCI-H660 cells represent a de novo NEPC model derived from a patient with small-cell carcinoma of prostatic origin, whereas LASCPC-01 cells represent a NEPC model established by propagating N-MYC/myrAKT1-driven tumor cells in vitro [62,63].

**Figure 1:**
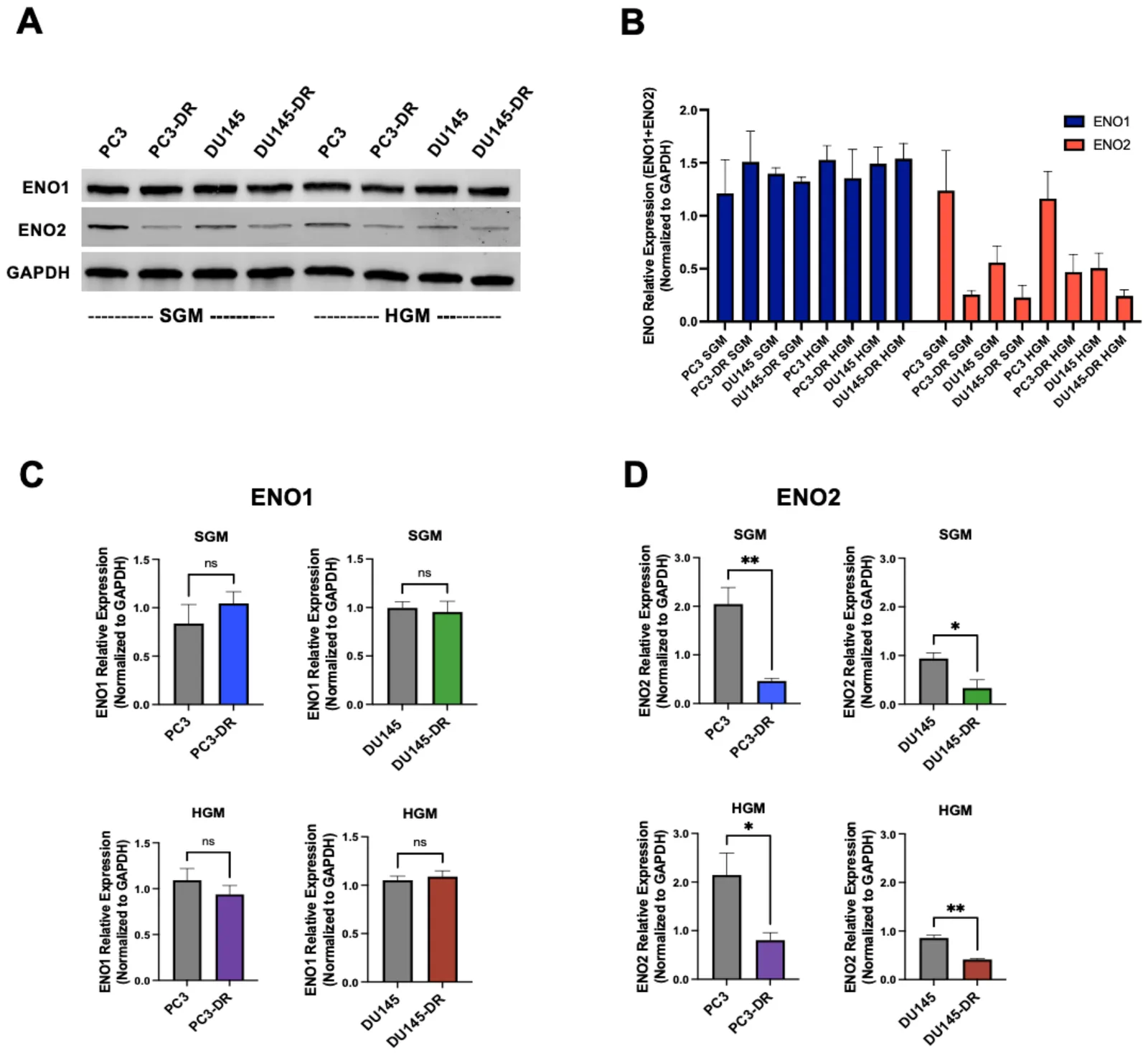
mCRPC cells exhibit elevated ENO1 protein expression, whereas ENO2 protein is markedly downregulated in DTX-resistant cells compared to sensitive cells. (**A**) Representative immunoblot showing protein expression of ENO1 (L-27 antibody) and ENO2 (10H7L13 antibody) in DTX-sensitive (PC3, DU145) and -resistant (PC3-DR, DU145-DR) mCRPC cell lines cultured in standard glucose medium (SGM) or high glucose medium (HGM). (**B**) Quantification of relative expression of ENO1 or ENO2 (ratio of individual protein expression divided by total ENO1 + ENO2 expression). (**C**,**D**) Quantification of ENO1 and ENO2 protein expression, respectively, in DTX-sensitive vs -resistant cells cultured in SGM or HGM. Quantified band values were obtained with Bio-Rad Image Lab software Version 6.1.0 Build 7 Standard Edition and plotted as relative protein expression normalized to GAPDH. Error bars represent mean ± standard error of the mean (SEM) from at least 3 independent experiments for each cell line. Statistical analysis was performed using unpaired two-tailed Student’s *t*-test. * *p* < 0.05, ** *p* < 0.01; ns = non-significant.

To assess the protein expression of ENO1 and ENO2, the mCRPC cell lines PC3, DU145, and their DTX-resistant variants PC3-DR and DU145-DR were cultured under standard (SGM) and high (HGM) glucose medium conditions. Immunoblotting analysis demonstrated that total ENO1 protein expression remained relatively uniform across DTX-sensitive and -resistant mCRPC cell lines cultured under both SGM and HGM conditions (Figure 1A–C). By contrast, total ENO2 protein was relatively less abundant than ENO1 particularly in DTX-resistant cells (Figure 1A,B).

Quantification of ENO1 protein expression for each individual cell line in relation to DTX-sensitivity vs resistance and SGM vs HGM revealed no significant differences between the comparison groups (Figure 1C). However, ENO2 protein expression was significantly reduced in DTX-resistant cells compared to their DTX-sensitive counterparts (Figure 1D). To rule out that these differences could be influenced by differential antibody reactivity, we validated antibody specificity by probing each antibody (ENO1; L-27 and ENO2; 10H7L13, both used at 1:2000 dilution) against recombinant human ENO1 and ENO2 in immunoblots. Each antibody detected only its corresponding enolase isoform, with no detectable cross-reactivity and with similar signal intensity against 0.1 µg of recombinant protein (Supplementary Figure S2).

At the transcript level we did not detect differences between ENO1 and ENO2 expression in DTX-sensitive cells (Supplementary Figure S3A). However, there were significant differences in DTX-resistant cells, with decreased ENO2 transcript expression compared to ENO1 in PC3-DR and DU145-DR cells grown in SGM (Supplementary Figure S3B). ENO1 transcript expression was significantly higher in PC3-DR cells compared to PC3 cells, but no significant difference was detected in the DU145/DU145-DR pair (Supplementary Figure S3C). By contrast, ENO2 transcript expression was downregulated in DTX-resistant cells compared to the sensitive cells (Supplementary Figure S3D), consistent with the protein expression results (Figure 1A,B,D).

Taken together, these results indicate that while the protein and transcript expression of ENO1 and ENO2, detected by immunoblotting and real time quantitative polymerase chain reaction (RT-qPCR), respectively, appears to be comparable in DTX-sensitive PC3 and DU145 cells, ENO2 expression is significantly less abundant in DTX-resistant cells.

### 2.2. ENO1 Is More Abundant than ENO2 on the Surface of NEPC Cells

**Figure 2:**
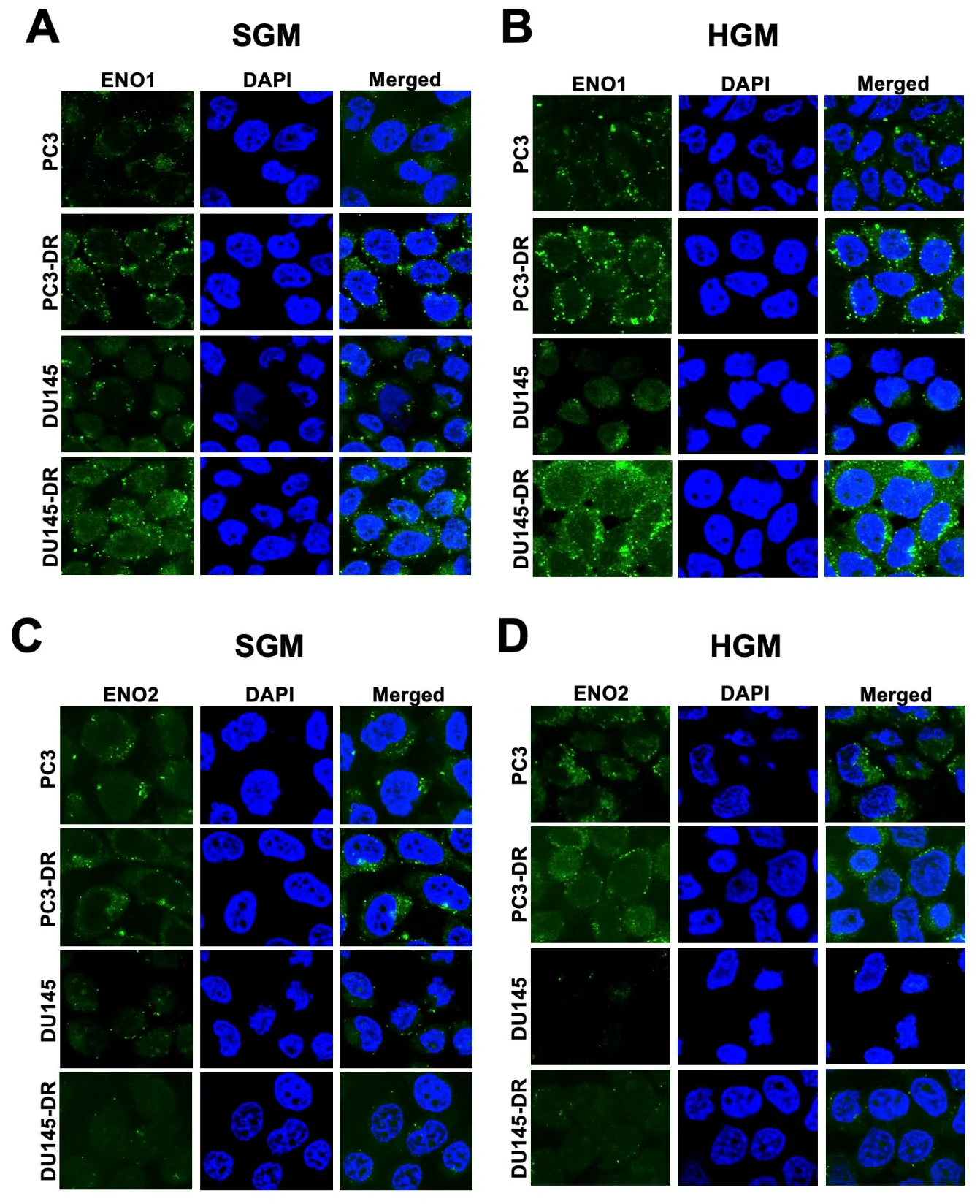
ENO1 is localized on the cell surface of DTX-resistant mCRPC cells. Confocal microscopy was performed to detect (**A**,**B**) ENO1 (antibody L-27) and (**C**,**D**) ENO2 (antibody 10H7L13) staining on the surface of PC3, PC3-DR, DU145, and DU145-DR mCRPC cell lines cultured in SGM and HGM.

Although ENO1 has been reported to translocate to the cell surface in several tumor types [24,25,26,40,64,65], its surface abundance in mCRPC and NEPC cells has not been clearly defined. Likewise, while ENO2 has been localized on the surface of neuroblastoma cells and brain tumors and implicated in metastatic cancers and NEPC progression [27,66,67,68], its cell surface abundance relative to that of ENO1 in PCa has not been fully characterized. To examine the surface localization of ENO1 and ENO2 in mCRPC cells, we first performed surface immunofluorescence staining followed by confocal microscopy visualization (Figure 2). Representative confocal imaging showed distinct surface ENO1 staining in DTX-resistant mCRPC cells compared to DTX-sensitive cells, cultured in SGM or HGM (Figure 2A,B). Surface ENO2 staining was less obvious in both DTX-sensitive and -resistant mCRPC cells regardless of the culture condition (Figure 2C,D).

**Figure 3:**
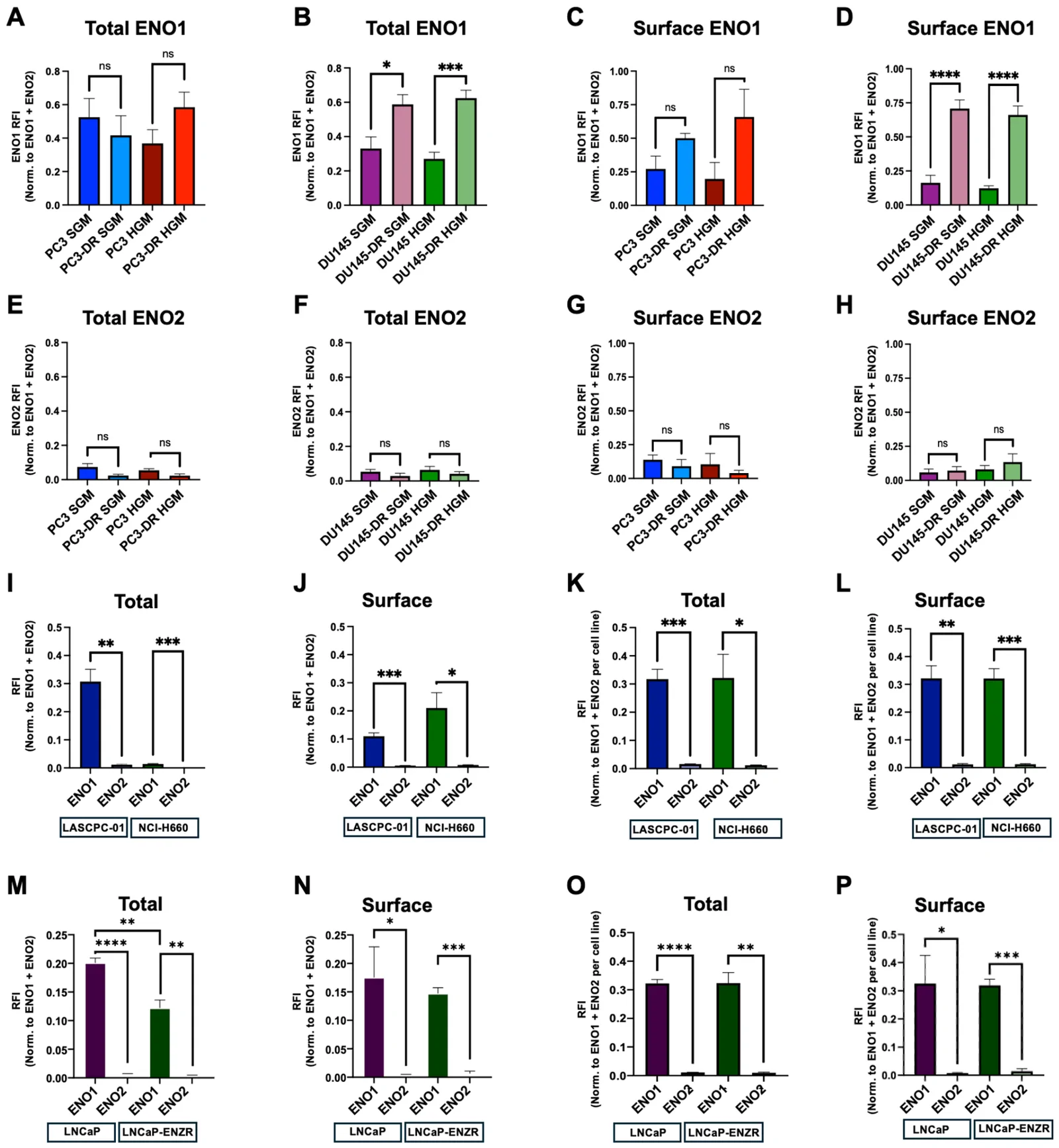
mCRPC and NEPC cells exhibit higher total and surface abundance of ENO1 compared to ENO2. Quantitative flow cytometric analysis was performed to determine the relative total and surface abundance of ENO1 (antibody L-27) and ENO2 (antibody 10H7L13). Relative fluorescence intensity (RFI) of total (**A**,**B**) and surface (**C**,**D**) ENO1 in mCRPC cells cultured in SGM and HGM. RFI of total (**E**,**F**) and surface (**G**,**H**) ENO2 in mCRPC cells cultured in SGM and HGM. RFI of total (**I**) and surface (**J**) ENO1 and ENO2 in the NEPC cell lines LASCPC-01 and NCI-H660. RFI of total (**K**) and surface (**L**) ENO1 and ENO2 in LASCPC-01 and NCI-H660 cells relative to each respective cell line. RFI of total (**M**) and surface (**N**) ENO1and ENO2 in LNCaP and LNCaP-ENZR cells. RFI of total (**O**) and surface (**P**) ENO1 and ENO2 in LNCaP and LNCaP-ENZR cells relative to each respective cell line. Data represent mean ± SEM from at least three independent flow cytometric experiments. Statistical significance was determined using an unpaired two-tailed Student’s *t*-test. * *p* < 0.05, ** *p* < 0.01, *** *p* < 0.001, **** *p* < 0.0001; ns = non-significant.

To validate these findings, we conducted flow cytometric studies to quantify both total and surface levels of ENO1 (L-27 antibody) and ENO2 (10H7L13 antibody) proteins in DTX-sensitive and -resistant cell lines. Compared to the PC3/PC3-DR pair (Figure 3A,C), DU145-DR cells displayed significantly increased relative ENO1 abundance, both total (Figure 3B) and on the cell surface (Figure 3D) compared to their DTX-sensitive counterparts under both SGM or HGM conditions. By contrast, the relative total (Figure 3E,F) and surface (Figure 3G,H) abundance of ENO2 in the four cell lines remained consistently low compared to those of ENO1. Consistent with the immunoblotting analyses, the relative levels of total ENO2 appeared to be lower in the DTX-resistant cell lines but were not significantly different compared to DTX-sensitive cells, regardless of whether cells were cultured in SGM or HGM (Figure 3E,F). Normalization of relative fluorescence intensity (RFI) values within each cell line further confirmed that total and surface ENO1 abundance were markedly elevated in DTX-resistant mCRPC cells grown under SGM or HGM conditions (Supplementary Figure S4A,B), whereas ENO2 abundance was decreased in DTX-resistant cells compared to the sensitive cells (Supplementary Figure S4C,D).

We extended our flow cytometry analysis to the NEPC cell lines NCI-H660 and LASCPC-01, observing significant differences between total (Figure 3I) and surface abundance (Figure 3J) of ENO1 and ENO2, with ENO1 consistently reaching significantly higher levels than ENO2 in the two cell lines. Normalization of RFI values within each cell line further confirmed that both total (Figure 3K) and surface (Figure 3L) abundance of ENO1 were significantly higher than those of ENO2, indicating that ENO1 is the predominant enolase isoform detectable on the surface of NEPC cells. We also examined the total and surface abundance of ENO1 and ENO2 in the AR+ LNCaP cell line and its enzalutamide-resistant counterpart, LNCaP-ENZR. Consistent with the results described above, the total (Figure 3M,O) and surface (Figure 3N,P) abundance of ENO1 were significantly higher than those of ENO2 in both cell lines. While LNCaP-ENZR cells exhibited a significant decrease in total ENO1 abundance compared to drug-sensitive LNCaP cells (Figure 3M), the cell surface abundance remained relatively similar in both cell lines (Figure 3N). Supplementary Figure S5 shows fluorescence intensity histograms corresponding to Figure 3 demonstrating signal distributions that corroborate the observed differences between total or surface ENO1 and ENO2 abundance.

A comparison of relative surface abundance of ENO1 and ENO2 in PC3 and DU145 DTX-sensitive and -resistant cells with NCI-H660 and LASCPC-01 cells revealed a pronounced increase in surface abundance of both glycolytic enzymes in the NCI-H660 and LASCPC-01 cell lines, albeit with ENO1 RFI values markedly higher than those of ENO2 (Supplementary Figure S6A,B). For additional validation of these flow cytometry results, we used a second set of specific antibodies against ENO1 (EPR19758) and ENO2 (E7D7I), confirming the significant increase in total and cell surface abundance of ENO1 compared to ENO2 in LASCPC-01 and NCI-H660 NEPC cells (Supplementary Figure S7). Representative gating strategies for surface and total ENO1 flow cytometry analyses are shown in Supplementary Figure S8.

Taken together, our flow cytometry findings indicate that total and surface abundance of ENO1 are significantly higher than those of ENO2 in mCRPC and NEPC cells.

### 2.3. ENO1 Knockdown Induces ENO2 Upregulation in DTX-Resistant mCRPC Cells

**Figure 4:**
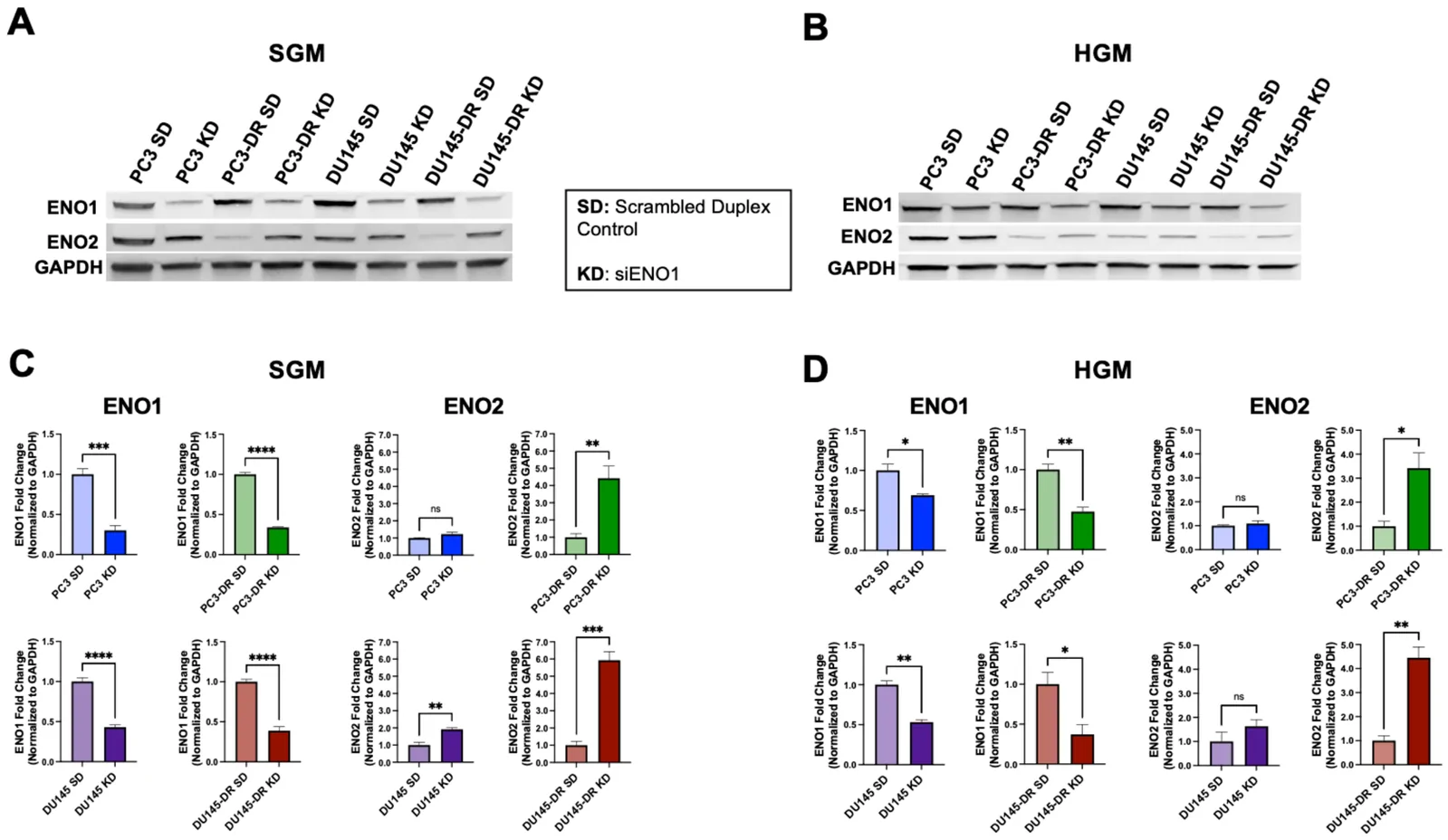
ENO1 knockdown increases ENO2 expression in docetaxel-resistant mCRPC cells. PC3, PC3-DR, DU145, and DU145-DR cells were transfected with ENO1-targeting siRNAs or scrambled duplex (SD) control oligos for 72 h. ENO1 knockdown efficiency was confirmed by immunoblotting in both (**A**) SGM and (**B**) HGM culture conditions. Reduced ENO1 (antibody L-27) protein expression with concomitant robust increase in ENO2 (antibody 10H7L13) expression was observed in DTX-resistant cells. (**C**,**D**). Quantification of ENO1 and ENO2 was performed for each cell line, and fold changes were calculated relative to SD controls. Data are presented as mean ± SEM from at least 3 independent blots for each cell line. Statistical significance was assessed using unpaired two-tailed Student’s *t*-test. * *p* < 0.05, ** *p* < 0.01, *** *p* < 0.001, **** *p* < 0.0001; ns = non-significant.

To further investigate the interplay between ENO1 and ENO2 in mCRPC cells, ENO1 was silenced in mCRPC cell lines cultured in SGM or HGM. Efficient ENO1 knockdown was confirmed by immunoblotting across all cell lines and culture conditions (Figure 4A,B). Interestingly, ENO1 depletion led to significant increase in ENO2 protein expression in PC3-DR and DU145-DR cells (Figure 4C,D). However, ENO2 protein upregulation in the DTX-sensitive parental lines was more limited, reaching statistical significance only in DU145 cells cultured in SGM (Figure 4C). These findings were confirmed at the transcript level (Supplementary Figure S9), consistent with a transcriptional compensatory mechanism occurring predominantly in DTX-resistant cells.

### 2.4. ENO1 Knockdown Reduces Proliferation and Clonogenicity in DTX-Resistant mCRPC Cells

**Figure 5:**
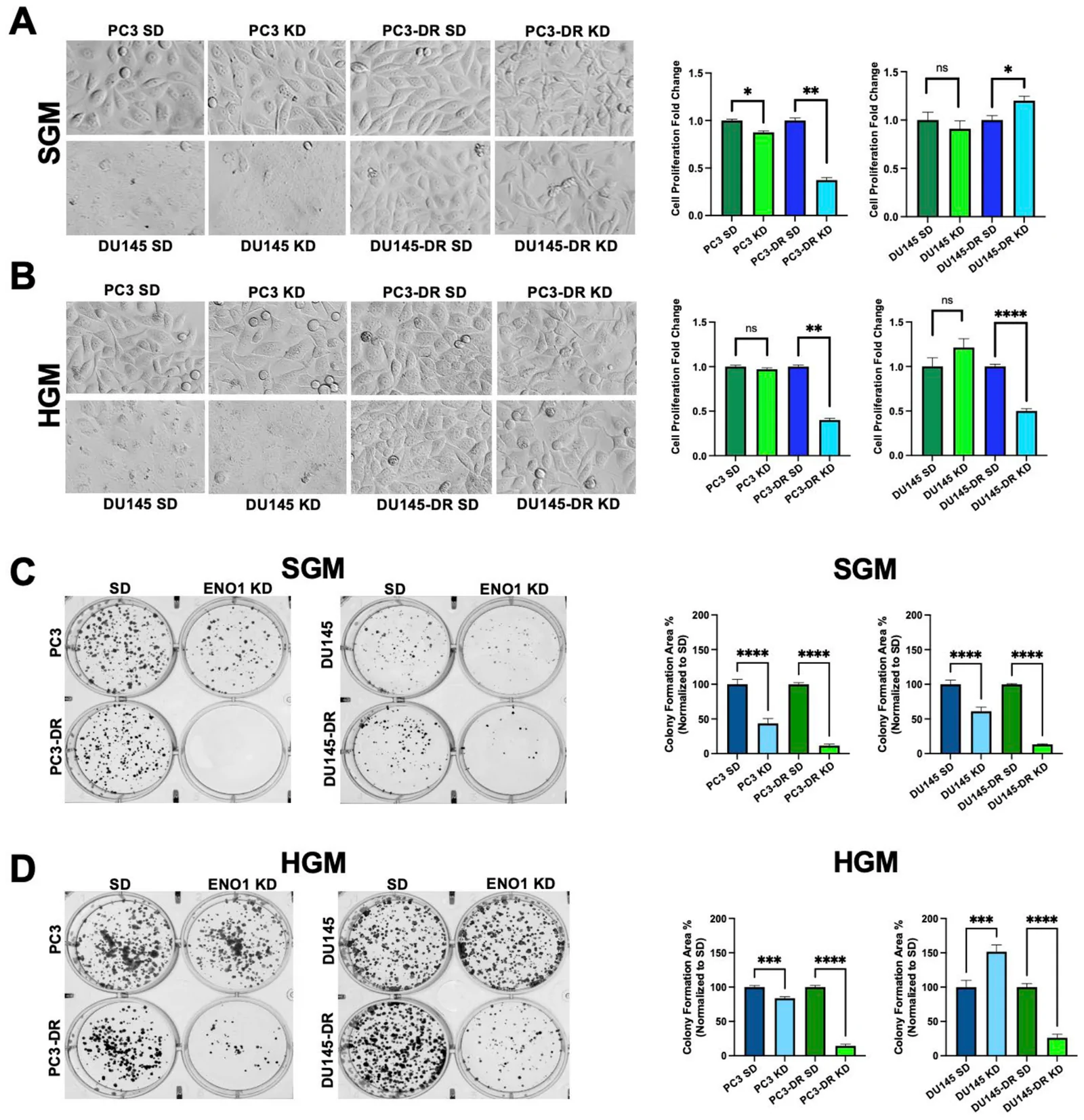
ENO1 knockdown reduces proliferation and clonogenicity in DTX-resistant mCRPC cells. (**A**,**B**) Hoffman Modulation Contrast (HMC) images and corresponding MTT (3-(4,5-dimethylthiazol-2-yl)-2,5-diphenyltetrazolium bromide) assay data for PC3, PC3-DR, DU145, and DU145-DR cells following ENO1 knockdown under SGM and HGM conditions. DU145 cells show a very flat morphology but develop an elongated, spindle-like shape upon transition to DTX resistance. Proliferation measurements are presented as mean ± SEM and normalized to SD controls. (**C**,**D**) Representative clonogenic assay images and quantification of colony formation in mCRPC cells following ENO1 knockdown under SGM and HGM conditions. Colonies were stained and analyzed after 14 days (SGM) or 10 days (HGM) of growth, and clonogenicity is expressed as the percentage of total area occupied by colonies (mean ± SEM), normalized to the corresponding SD control. Statistical significance for all quantitative comparisons was determined using a two-tailed Student’s *t*-test; * *p* < 0.05, ** *p* < 0.01, *** *p* < 0.001, **** *p* < 0.0001; ns = non-significant.

We next assessed the impact of ENO1 knockdown on mCRPC cell proliferation and death, evaluating first the morphology of ENO1-depleted DTX-sensitive and -resistant mCRPC cell lines cultured in SGM (Figure 5A) or HGM (Figure 5B). While DTX-resistant cells exhibited subtle morphological changes post-knockdown, there was no visual evidence of abundant cell death. We measured cell proliferation 72 h after ENO1 knockdown, observing a robust and significant reduction in DTX-resistant cells under both SGM and HGM conditions (Figure 5A,B, respectively). However, this reduction was not observed in DU145-DR cells grown in SGM; instead, these cells showed a slight but significant increase in proliferation compared to SD controls. These results suggest that total ENO1 is required for maintaining the proliferative capacity of DTX-resistant mCRPC cells. ENO1 knockdown also impaired the clonogenic capacity of DTX-sensitive and -resistant PC3 and DU145 cells cultured in SGM (Figure 5C) and HGM (Figure 5D). Notably, cells grown in HGM formed denser colonies than cells grown in SGM, indicating a clonogenic growth advantage under high-glucose conditions. Quantitative analysis performed 10 days (HGM) or 14 days (SGM) post-seeding showed a marked reduction in clonogenicity following ENO1 knockdown, measured as the percentage of area covered by colonies and normalized by standard deviation. This effect was more pronounced in DTX-resistant cells (Figure 5C,D). The decrease in colony formation was consistent across both SGM and HGM culture conditions, highlighting the critical role of ENO1 in maintaining the proliferative and survival capacity of DTX-resistant mCRPC cells.

We also evaluated the impact of ENO1 depletion on the sensitization of PC3-DR and DU145-DR cells to the anti-proliferative effects of DTX. After treatment with increasing concentrations of DTX (10, 20, 40, 60, 100 nM), ENO1-depleted cells did not exhibit significant increases in DTX sensitivity compared to their respective SD controls (Supplementary Figure S10A,B), suggesting that despite reduction in clonogenic capacity, ENO1 knockdown appeared insufficient to restore this sensitivity. However, we cannot rule out that the very low numbers of colonies induced by the ENO1 knockdown precluded an accurate quantification of the sensitization effect.

### 2.5. ENO1 Surface Targeting Inhibits Proliferation, Clonogenic Capacity, and Tumorsphere Formation in mCRPC and NEPC Cell Lines

**Figure 6:**
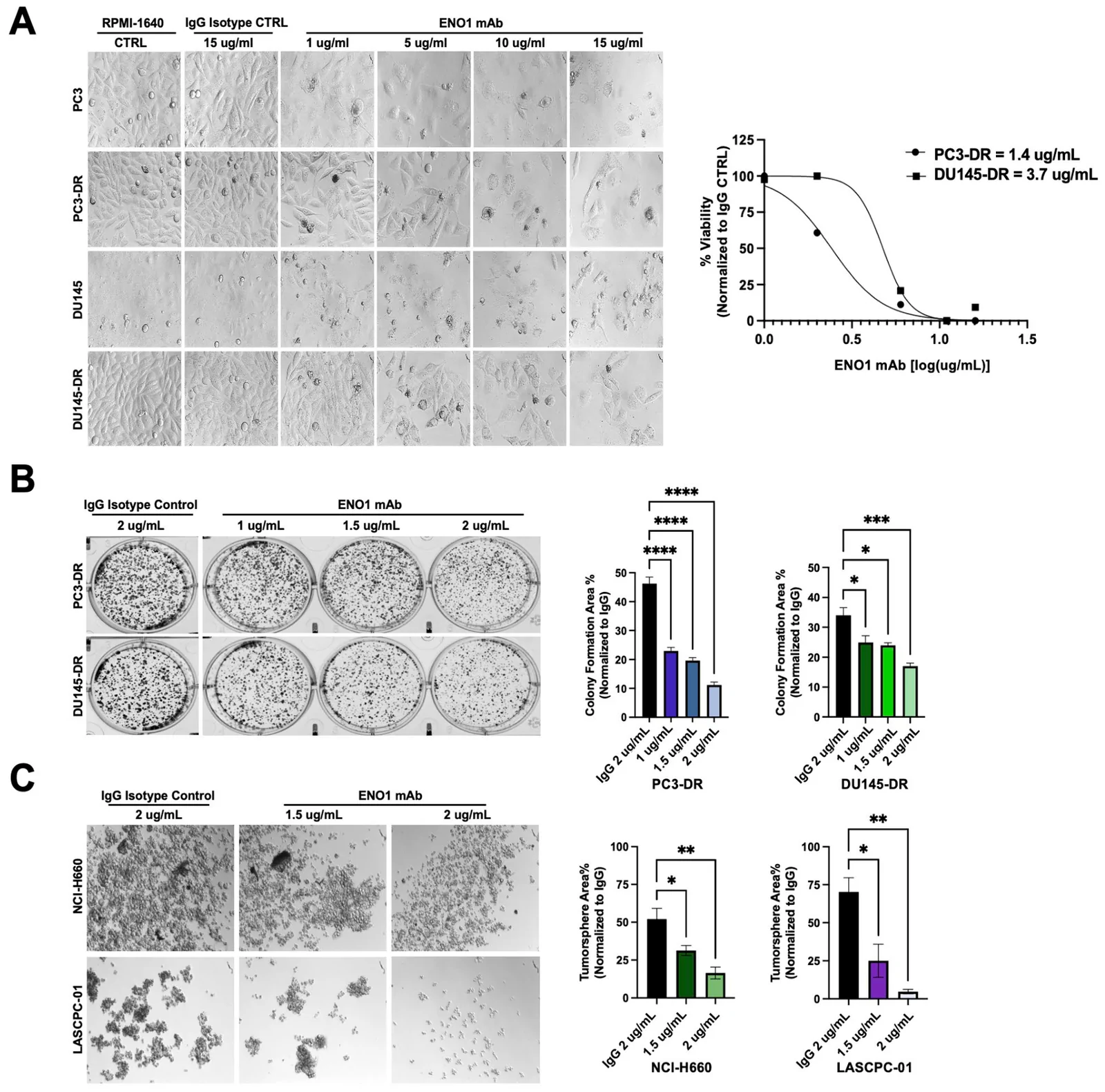
Treatment with ENO1 monoclonal antibody (mAb) alters cell morphology and suppresses clonogenic and tumorsphere capacity in DTX-resistant mCRPC and NEPC cells. Following treatment with ENO1 mAb (**A**) cell morphology was assessed by HMC microscopy (left panel). Cells were stained with Crystal Violet and IC_50_ values were calculated by nonlinear regression using a variable-slope sigmoidal model in GraphPad Prism (right panel); (**B**) clonogenic potential of mCRPC cells was evaluated after 10 days of growth colony and quantified as the percentage of total area occupied by colonies; and (**C**) tumorsphere-forming capacity of NEPC cells was assessed after 7 days of growth. Data are presented as mean ± SEM, normalized to corresponding isotype control. Statistical significance was determined using a one-way ANOVA. * *p* < 0.001, ** *p* < 0.0001. *** *p* < 0.001, **** *p* < 0.0001.

Since ENO1 knockdown reduced PCa cell proliferation and clonogenic capacity, we next sought to determine if antibody targeting of cell surface ENO1 would produce similar effects. For these studies, DTX-sensitive and -resistant PC3 and DU145 cells were treated with increasing concentrations of ENO1 mAb (L-27; 1, 5, 10, and 15 µg/mL) or mouse IgG isotype control (15 µg/mL) for 72 h (Figure 6A). Microscopic visualization showed concentration-dependent morphological changes, including evidence of cell death, in cells treated with ENO1 mAb compared to cells treated with IgG isotype control (Figure 6A, left panel). Consistent with this observation, cell viability was reduced after ENO1 mAb treatment (Supplementary Figure S11A). IC_50_ values calculated for the ENO1 mAb treatments in the DTX-resistant cells were approximately 1.4 µg/mL for PC3-DR and 3.7 µg/mL for DU145-DR (Figure 6A, right panel). In equivalent experiments with ENO2 mAb (10H7L13; 1, 5, 10, and 15 µg/mL), neither cell viability nor cell morphology were affected (Supplementary Figure S11B,C). Hence, subsequent experiments were conducted only with ENO1 mAb.

To determine if ENO1 surface targeting affected clonogenic capacity, DTX-resistant cells were treated with 1, 1.5 and 2 µg/mL of ENO1 mAb or IgG isotype control at 2 µg/mL for 10 days (Figure 6B, left panel). ENO1 mAb treatment produced a significant, concentration-dependent decrease in colony formation compared to the isotype control (Figure 6B, right panel). Because NCI-H660 and LASCPC-01 NEPC cells grow in suspension and are not suitable for clonogenic assays, we used a tumorsphere formation assay to evaluate the effect of ENO1 surface targeting on their tumorigenic capacity. Cells were treated with 1.5 and 2 µg/mL of ENO1 mAb or IgG isotype control at 2 µg/mL for 7 days, and results demonstrate that both concentrations significantly reduced tumorsphere formation compared to the IgG isotype control (Figure 6C).

Taken together, these results indicate that antibody-mediated targeting of surface ENO1 reduces proliferation, clonogenic survival, and tumorigenic capacity in mCRPC and NEPC cells, further supporting ENO1 as a viable cell-surface therapeutic target in advanced PCa.

### 2.6. ENO1 Surface Targeting Induces DNA Damage, Cell Cycle Perturbations, and Apoptosis in DTX-Resistant PCa Cells

**Figure 7:**
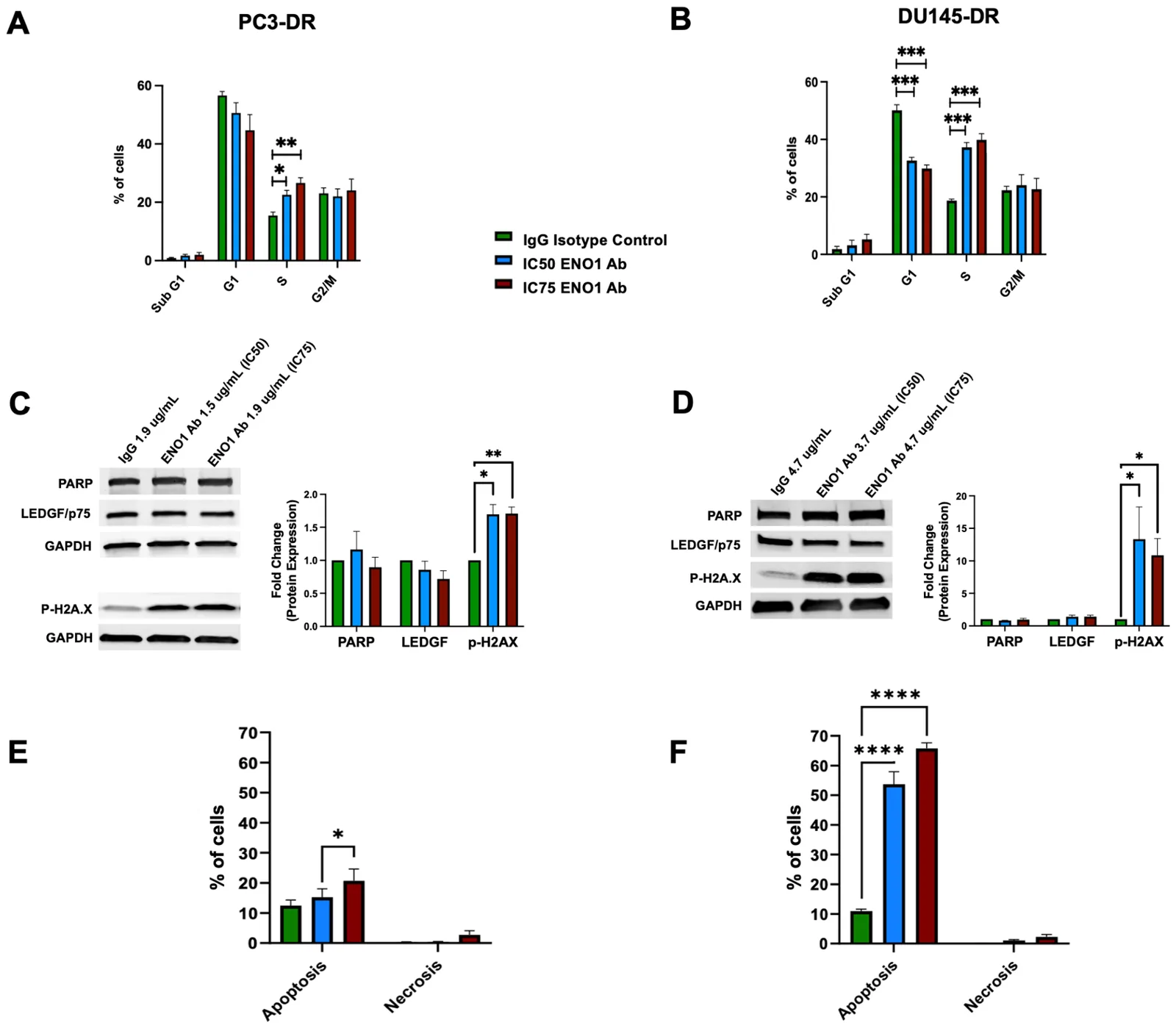
Treatment with anti-ENO1 mAb perturbs cell cycle progression, induces S-phase accumulation, and promotes apoptosis in mCRPC cells. Following treatment with ENO1 mAb, cell cycle distribution of (**A**) PC3-DR cells and (**B**) DU145-DR cells was analyzed by flow cytometry using propidium iodide (PI) staining. (**C**,**D**) Expression and quantification of the DNA damage marker p-H2A.X and the DNA damage repair proteins PARP and LEDGF/p75 was assessed by immunoblotting; and (**E**,**F**) the percentages of apoptotic and necrotic cells were determined by Annexin V/PI staining and flow cytometry. Data are presented as mean ± SEM. Statistical significance was determined using a one-way ANOVA. * *p* < 0.05, ** *p* < 0.01, *** *p*< 0.001, **** *p* < 0.0001.

To gain mechanistic insights into how ENO1 surface targeting reduces proliferation, clonogenic growth, and tumorsphere formation, we performed flow cytometric cell cycle analysis on DTX-resistant PC3-DR and DU145-DR cells treated with ENO1 mAb for 72 h using concentrations corresponding to IC_50_ and IC_75_ values (Figure 7).

These experiments showed a significant increase in the S-phase cell population, accompanied by a non-significant decrease in the G_1_ fraction, suggesting that ENO1 surface targeting impairs S-phase progression and may induce replication stress (Figure 7A,B). To determine if these changes were associated with DNA damage and cell death, we first assessed by immunoblotting the expression of p-H2A.X, a cellular sensor of DNA double-strand breaks, and the DNA repair proteins poly-ADP-ribose polymerase (PARP) and lens epithelium-derived growth factor p75 (LEDGF/p75) [69]. The results showed a significant increase in p-H2A.X after ENO1 antibody treatment compared with IgG isotype controls, while LEDGF/p75 and PARP levels remained unchanged (Figure 7C,D). The p-H2A.X increase was more robust in DU145-DR cells (Figure 7D). To measure cell death, we performed Annexin V/7-AAD staining after ENO1 mAb treatment and observed a concentration-dependent increase in Annexin V-positive, 7-AAD-negative apoptotic cells, with no increase in 7-AAD-positive necrotic cells, suggesting that ENO1 surface inhibition primarily drives apoptotic death in DTX-resistant mCRPC cells (Figure 7E,F). This increase in apoptosis was also more robust in DU145-DR cells (Figure 7F).

### 2.7. ENO1 Transcript Expression Is Higher than ENO2 Expression in Prostate Tumor Tissues

**Figure 8:**
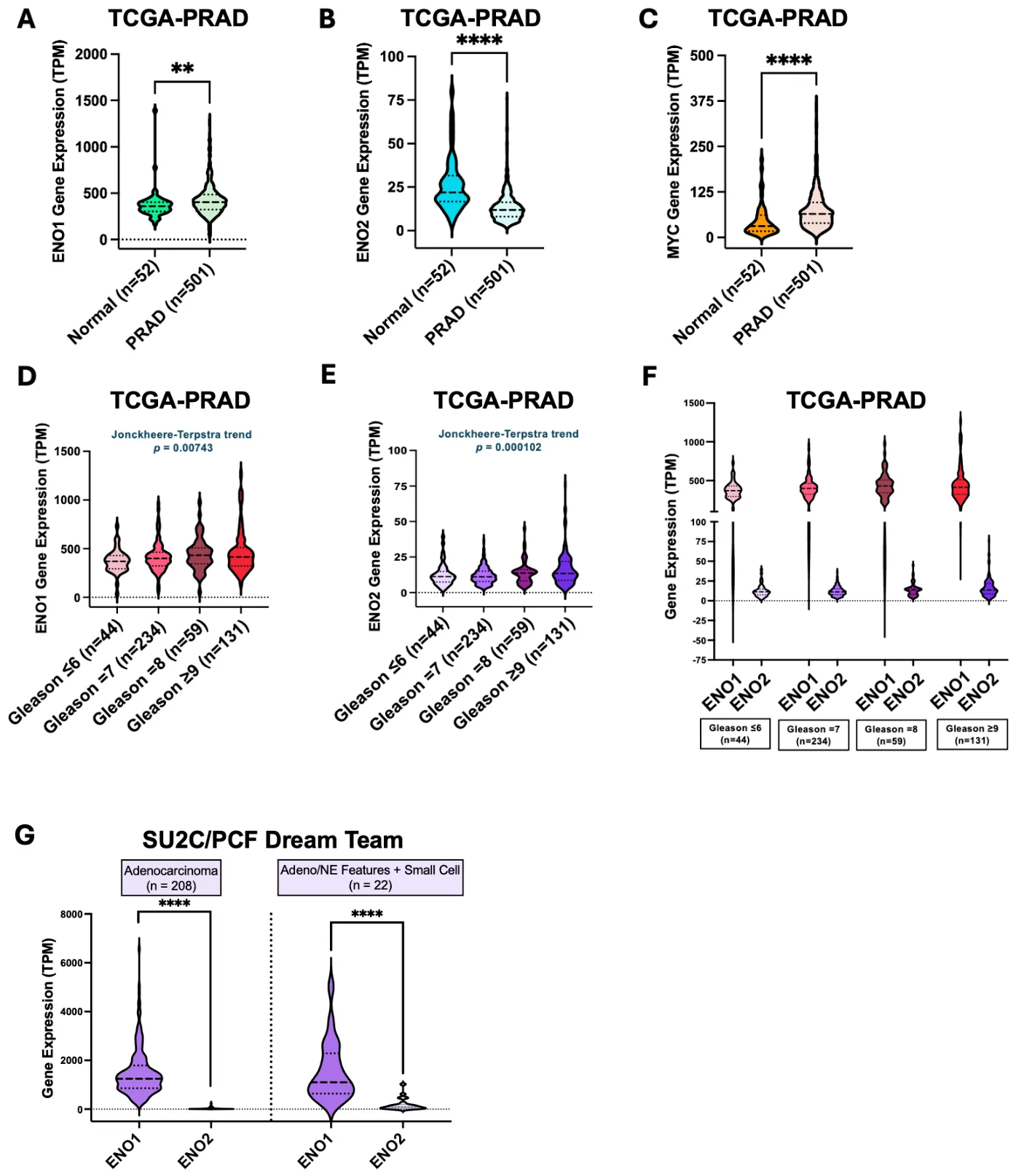
ENO1 transcript expression is more abundant than ENO2 expression in clinical primary prostate tumors. (**A**) *ENO1* transcript expression is significantly higher in prostate adenocarcinoma (PRAD) samples than in normal prostate tissues. (**B**) ENO2 transcript expression is significantly downregulated in PRAD samples compared to normal prostate tissues. (**C**) MYC transcript expression is significantly higher in PRAD samples than in normal prostate tissues. (**D**–**F**) ENO1 and ENO2 transcript expression increased with Gleason score in prostate tumors (Jonckheere-Terpstra test for trend; ENO1, *p* = 0.0074; ENO2, *p* = 0.0001). (**G**) ENO1 and ENO2 transcript expression in the SU2C/PCF Dream Team tumor samples, stratified as adenocarcinoma and adeno/NE features + small-cell carcinoma. ENO1 was significantly higher than ENO2 in both subgroups**.** Data did not follow a normal distribution, as determined by the Shapiro-Wilk normality test. Therefore, differences in transcript expression between normal and tumor tissues were analyzed using the nonparametric Mann-Whitney U test for independent samples, whereas monotonic trends across increasing Gleason scores were evaluated using the Jonckheere-Terpstra trend test (*p* = 0.00743 for panel (**D**) and *p* = 000102 for panel (**E**)). ENO1 and ENO2 transcript expression was compared within each histologic subgroup using the Wilcoxon signed-rank test for paired samples. Data are presented as medians with interquartile ranges (IQR). ** *p* < 0.01, **** *p* < 0.0001.

We next examined the transcript expression of *ENO1* and *ENO2* genes in clinical prostate tumors using publicly available data from the TCGA-PRAD cohort. Prostate tumor tissue samples showed significantly higher transcript levels of *ENO1* and *ENO2* compared to normal prostate tissues (Figure 8A,B). The *ENO1* transcriptional regulator *MYC* also showed higher transcript levels in the tumor tissues (Figure 8C).

Violin plots demonstrated increased median transcripts per million (TPM) values and broader distributions for *ENO1* (normal TPM: 360; tumor TPM: 403.6) and *MYC* (normal TPM: 31.25; tumor TPM: 64.60) in primary PCa samples, whereas *ENO2* exhibited much lower median values (normal TPM: 21.97; tumor TPM: 11.88) and a compressed distribution in tumors relative to normal tissues (Figure 8A–C). *ENO1* median transcript expression increased with Gleason score (Jonckheere-Terpstra test for trend, *p* = 0.0074) (Figure 8D), as did *ENO2* median transcript expression (*p* = 0.00010) (Figure 8E). Notably, *ENO1* expression levels were substantially higher than *ENO2* across all the Gleason score groups (Figure 8F). In the SU2C/PCF Dream Team cohort, which includes adenocarcinomas, adenocarcinomas with neuroendocrine features (adeno/NE features), and small-cell carcinoma, *ENO1* transcript expression was also significantly higher than *ENO2* in both the adenocarcinoma and the combined adeno/NE features + small-cell subgroups (Figure 8G), indicating that ENO1 is the most abundant enolase isoform in advanced tumors with neuroendocrine features. 

## 3. Discussion

Although most mCRPC tumors remain AR-driven, prolonged AR signaling inhibition can select for drug-resistant, AR-independent, and NEPC-like states characterized by PSMA downregulation, molecular heterogeneity, and lineage plasticity [4,10,11,15,16,17,18,19,22,23,70]. Because PSMA-low mCRPC and NEPC currently lack effective targetable antigens, identifying alternative cell-surface proteins for theranostic or therapeutic approaches remains critical [4,16,23]. Here we examined the total and surface abundance of ENO1 and ENO2 in mCRPC and NEPC cellular models, evaluated the effects of ENO1 knockdown and mAb targeting on cancer cell properties, and analyzed ENO1 and ENO2 transcript expression in clinical tumors. Our study revealed several key findings: (1) ENO1 is more abundant than ENO2 on the surface of DTX-resistant mCRPC cells and NEPC cells; (2) DTX-resistant mCRPC cells show comparable ENO1 expression but marked ENO2 downregulation; (3) ENO1 knockdown reduces mCRPC cell proliferation and clonogenicity despite ENO2 upregulation, suggesting a potential compensatory response; (4) mAb-mediated targeting of surface ENO1 leads to decreased proliferation, cell cycle perturbations, increased p-H2A.X, and apoptosis; and (5) clinical primary prostate tumors, including high-Gleason tumors and tumors with NE features, express significantly higher levels of ENO1 than ENO2. Together, these findings identify ENO1 as a functionally relevant and accessible cell-surface candidate target in advanced PCa. They also suggest that mCRPC and NEPC cells, particularly DTX-resistant cells, undergo metabolic and phenotypic reprogramming that favors ENO1-driven proliferation and metastasis during therapy resistance and tumor progression. Consistent with this, our previous work [71] linked DTX-resistance in PC3 and DU145 cells to cancer stem-cell, EMT, and metabolic/energy associated gene programs implicated in advanced mCRPC and NEPC [19,70].

We did not observe significant differences in ENO1 or ENO2 total expression or surface abundance in mCRPC cells cultured in SGM (11.1 mM) or HGM (25 mM), suggesting that this range of glucose availability is still sufficient to drive comparable protein levels. However, increased ENO1 expression concomitant with ENO2 downregulation in DTX-resistant mCRPC cells suggests a potential link between therapy resistance and metabolic or membrane adaptations that may facilitate ENO1 surface accumulation. Although ENO2 surface localization has been reported in neuronal cells and brain tumors, it remains poorly defined in non-neuronal cancers [27,67,68,72,73,74,75]. By contrast, surface ENO1 is well established in multiple cancer types and associated with cell proliferation, migration, invasion, and metastasis [25,26,39,76].

Surface ENO1 functions as a plasminogen receptor, promoting plasmin generation, extracellular matrix degradation, migration, invasion, and metastasis [40,41,77,78,79]. A previous report indicated that antibody targeting of ENO1 in PC3 and DU145 cells inhibited inflammation-enhanced migration and chemotaxis, osteoclast differentiation, and inflammatory cytokine secretion [80]. A functional link between surface ENO1 and glycolysis has also been demonstrated. For instance, cell surface ENO1 interacts directly with the lactate exporter monocarboxylate transporter 4 (MCT4) and its blockade with antibodies reduced surface MCT4, extracellular lactate accumulation after irradiation, and glycolysis-related gene sets in colorectal cancer models [81]. In multiple myeloma cells, recombinant human ENO1 protein dose-dependently increased secreted lactate and intracellular lactate dehydrogenase (LDH) activity and upregulated hypoxia inducible factor 1 alpha (HIF-1α), hexokinase 2 (HK2), and glucose transporter 1 (GLUT1) [44]. These effects were reproduced with catalytically inactive ENO1 mutants but attenuated by a humanized anti-ENO1 antibody, indicating that extracellular ENO1 promotes glycolysis independent of its catalytic activity [44]. Whether surface ENO1 similarly regulates lactate export or glycolytic gene expression in PCa cells remains to be determined.

ENO1 trafficking to the cell surface has been documented in bladder [82], breast [83,84], lung [85], and pancreatic cancers [40] where cellular stress and growth factor signaling can regulate its surface localization. For example, in breast cancer, heat shock protein 70 (Hsp70) promotes membrane ENO1 localization following epidermal growth factor (EGF) or lipopolysaccharide (LPS) stimulation [83], while store-operated calcium entry (SOCE) mediated by stromal interaction molecule 1 (STIM1) and the calcium channel protein 1 (ORAI1) regulates ENO1 externalization [64]. Additionally, ENO1 surface translocation and exosomal secretion may facilitate integrin-associated signaling, EMT, migration, invasion, and metastasis in liver cancer [42]. Although these mechanisms have not been established in mCRPC or NEPC, both disease states exhibit cellular stress and activation of growth factor signaling pathways regulating cell cycle progression, survival, and migration [4,86,87,88]. Post-translational modifications (PTMs), including methylation, phosphorylation, and acetylation, may also contribute to ENO1 subcellular localization in tumor cells [65,89,90,91].

ENO1 knockdown significantly decreased cell proliferation and clonogenic capacity in mCRPC cell lines. Although ENO2 expression was upregulated in these cells following ENO1 depletion, it did not fully compensate for the loss of ENO1. For instance, while DU145-DR cells retained high ENO2 expression and proliferative capacity following ENO1 knockdown (Figure 4D, Figure 5A and Figure S9), the clonogenic capacity of both PC3-DR and DU145-DR cells was markedly impaired after ENO1 knockdown (Figure 5C,D). These results suggest that ENO2 upregulation under our experimental conditions provides incomplete, context-dependent compensation for ENO1 depletion and may be insufficient to fully restore glycolytic flux or proliferative capacity. In a high glucose tumor microenvironment (TME), ENO2 may complement ENO1 glycolytic and proliferative functions, consistent with its association with metastatic progression in PCa, including NEPC [66] and other cancer types [92,93,94,95,96]. Whether this response involves altered glycolytic activity, non-glycolytic signaling, or surface localization remains unclear. Direct ENO2 perturbation and evaluation of the effects in docetaxel-resistant xenograft models will be needed in future studies to determine its contribution to tumor growth in the resistant state. Prior associations of ENO2 with metastatic progression in PCa and other cancers provide a rationale for these investigations. Together, these findings suggest that ENO1, and perhaps to a lesser extent ENO2, support mCRPC cell proliferation and clonogenic growth and may contribute to their aggressive phenotype, particularly in glucose-rich TMEs.

While we assessed whether ENO1 knockdown enhanced mCRPC sensitivity to DTX in clonogenic assays, we did not detect increased DTX responses in ENO1-depleted PC3-DR and DU145-DR cells (Supplementary Figure S10). One possible explanation is that ENO1 depletion reduces the actively proliferating cell fraction, leaving fewer cells and colonies susceptible to DTX, a taxane drug that stabilizes microtubules and induces mitotic arrest and apoptosis preferentially in rapidly cycling cells [9]. These results are in contrast with reports of ENO1 depletion enhancing responses to non-taxane drugs in other tumor types [46,48,50,53,97,98].

Our results also showed that treatment with anti-ENO1 monoclonal antibodies reduced proliferation, clonogenic growth, and tumorsphere formation in mCRPC and NEPC models, including DTX-resistant cells, consistent with a report showing that antibody-mediated blockade of cell surface ENO1 attenuated PC3-derived xenograft growth and inflammation-induced migration in PC3 and DU145 cells [80]. These results were broadly consistent with those of ENO1 knockdown on clonogenic and tumorsphere capacity, although the two approaches did not fully overlap for bulk proliferation in every cell line. They also extend prior studies showing that ENO1-directed antibody targeting impairs cancer cell glycolysis, invasion, tumor growth, and TME-driven progression in other cancer types [40,44,76,81]. To our knowledge, the anti-ENO1 monoclonal antibody effects on proliferation, clonogenic growth, and tumorsphere formation have not been previously reported in DTX-resistant mCRPC or NEPC cells, making these observations particularly relevant in the context of treatment-resistant advanced PCa. Interestingly, the lack of an anti-proliferative effect with ENO2 antibody treatment (Supplementary Figure S11B,C) is consistent with our flow cytometric data (Figure 3) demonstrating more abundant ENO1 on the surface of mCRPC and NEPC cells than ENO2.

Interestingly, antibody targeting of surface ENO1 also significantly affected cell-cycle progression, DNA integrity, and cell survival. We observed a trend toward G1 arrest and significant S-phase accumulation, accompanied by increased p-H2A.X, a marker of replication-associated DNA double-strand breaks (DSBs) [99]. Strikingly, LEDGF/p75 and PARP, proteins linked to DSB repair [69], showed no detectable increase under these conditions, raising the possibility that DNA damage accumulates without a corresponding repair response during short-term surface ENO1 targeting. Whether this contributes to the observed loss of clonogenic and tumorsphere potential remains to be determined. Although increased γ-H2AX following ENO1 targeting is consistent with DSB induction, it may also reflect replication stress or apoptosis-associated DNA fragmentation. Future kinetic studies examining the appearance of γ-H2AX relative to apoptotic signaling will help distinguish between these possibilities and define the mechanistic consequences of ENO1 inhibition. Together, these results support the notion that ENO1 surface targeting results in DNA damage and promotes a shift from survival to apoptosis in PCa cells. 

Several mechanisms may underlie the reduction in proliferation, clonogenicity, tumorsphere capacity, and DNA integrity observed after ENO1 mAb treatment. These include disruption of glycolysis required for proliferation, including interference with glycolytic signaling complexes and lactate export via interactions with transporters such as MCT4 [33,35,37,38]; blockade of ENO1 plasminogen-receptor functions and associated invasion niches, limiting matrix remodeling and disrupting ENO1-dependent adhesion and migration in the TME [40,41,79,83,84,85]; perturbation of stemness and self-renewal signaling networks, including the PI3K/AKT/mTOR pathway, and ENO1-containing membrane and extracellular-vesicle complexes that reinforce survival and self-renewal [35,37,38,42,44,45,53,77,89]; and attenuation of ENO1-driven transcriptional programs maintaining cancer stem-like cells. These signaling pathways combined with impaired glycolysis may induce cellular stress and DNA damage without a rapid and robust repair response, reducing proliferative and clonogenic potential [26,39,99,100].

While our immunoblotting and RT-qPCR results did not establish an absolute abundance of ENO1 over ENO2, except for the significant ENO2 downregulation in DTX-resistant cells, our flow cytometry studies using two different sets of validated ENO1 and ENO2 antibodies (Figure 3 and Supplementary Figure S7) did support a higher abundance of ENO1 over ENO2, both total and on the surface of mCRPC and NEPC cells. These differences could be due to the higher sensitivity of the flow cytometry method for detecting protein abundance. Significantly higher *ENO1* upregulation, compared to ENO2, was also observed in clinical prostate tumors with high Gleason score from The Cancer Genome Atlas-Prostate Adenocarcinoma (TCGA-PRAD) dataset, which supports its standing as a promising therapeutic target. Although *ENO2* tumor abundance was significantly lower than *ENO1* in these clinical samples, it also showed an increasing trend with higher Gleason scores, suggesting that both enolase isoforms are associated with prostate tumor progression despite differential expression levels. However, the lower *ENO2* abundance relative to *ENO1* in the TCGA-PRAD and Stand Up to Cancer/Prostate Cancer Foundation (SU2C/PCF) PCa cohorts aligns with our flow cytometry data. Elevated *ENO1* and *MYC* expression in primary tumors is also consistent with *MYC*-mediated *ENO1* transactivation, which drives glycolytic reprogramming and tumor progression and aggressiveness [31]. These observations support reports associating ENO1 overexpression with tumor aggressiveness and poor clinical outcomes in other cancer types [54,55,56,82].

Two limitations of our study must be acknowledged. First, although we observed significant differences between *ENO1* and *ENO2* expression in the SU2C/PCF metastatic PCa cohort, the number of samples with neuroendocrine features or small-cell histology was limited, guaranteeing future studies with larger cohorts. Second, while treatment with the anti-ENO1 mAb phenocopied several ENO1-knockdown effects, we did not establish that its activity was mediated exclusively by surface-exposed ENO1. Therefore, we cannot exclude antibody internalization or engagement of secreted ENO1. Addressing these limitations will be important for advancing ENO1 as a theranostic target for advanced PCa and other tumors.

In summary, together with established roles of ENO1 in tumor cell invasion, migration, and metastasis, our observations provide a strong rationale for its therapeutic targeting in mCRPC and NEPC. ENO1 should be viewed not only as a biomarker of aggressive PCa but also as a therapeutically accessible cell surface antigen with broad translational potential for treatment-refractory PCa. Robust ENO1 expression and surface abundance on PSMA-low mCRPC and NEPC cells supports the development of ENO1-directed therapeutic strategies, including ENO1-based radioligand therapy for patients that are ineligible for [^177^Lu]-PSMA due to PSMA downregulation, ENO1 antibody-based immunotherapy, antibody-drug conjugates, chimeric antigen receptor CAR-T/CAR-NK cell therapies, in combination with standard-of-care treatments and DNA-damaging drugs.

## 4. Materials and Methods

### 4.1. Cell Lines and Culture 

The PCa cell lines PC3, DU145, LNCaP, and 22RV1 were acquired from the American Type Culture Collection (ATCC, Manassas, VA, USA; Cat: ATCC-CRL-1435, ATCC-HTB-81, ATCC-CRL-1740, ATCC-CRL-2505, respectively) and cultured in RPMI-1640 medium. Cells were cultured in standard glucose medium (SGM, 11.1 mM) (Genesee Scientific, El Cajon, CA, USA; Cat: 30-2001) or high glucose medium (HGM, 25 mM) (ATCC; Cat: 30-2001) supplemented with 10% fetal bovine serum (FBS) (Genesee Scientific; Cat: 25-550), 1% penicillin/streptomycin (pen/strep) (Corning, Glendale, AZ, USA; Cat: 30-002-CI), and Normocin 1G (Invivogen, San Diego, CA, USA; Cat: NC9390718). The NCI-H660 cell line was acquired from ATCC (Cat: ATCC-CRL-5813) and cultured in HGM HITES medium containing 5% FBS, 1% pen/strep, 2mM L-glutamine (Corning, Cat: 25-005-CI), 0.005 mg/mL insulin (Sigma-Aldrich, St. Louis, MO, USA; Cat: I0516), 0.01 mg/mL transferrin (Sigma-Aldrich; Cat: T1147), 30 nM sodium selenite (Sigma-Aldrich, Cat: S9133), 10 nM hydrocortisone (Sigma-Aldrich, Cat: H0396), and 10 nM beta-estradiol (Sigma-Aldrich; Cat: E8875). The LASCPC-01 cell line was acquired from ATCC (Cat: ATCC-CRL-3356) and cultured in HGM modified HITES medium (30 nM sodium selenite, 10 nM beta-estradiol, 0.005 mg/mL insulin, and 2 mM of L-glutamine) supplemented with 5% FBS, 1% pen/strep, 10 nM hydrocortisone, and 0.01 mg/mL transferrin. The MDA-PCa-2b cell line (ATCC, Cat: CRL-2422) was cultured in F-12K basal medium (Corning, Cat: 10-025-CV) supplemented with 20% FBS, 1% penicillin–streptomycin, 25 ng/mL cholera toxin (Sigma-Aldrich, Cat: 8052), 10 ng/mL human epidermal growth factor (Sigma-Aldrich, Cat: E9644), 5 µg/mL insulin, 100 pg/mL hydrocortisone, 45 nM selenious acid (Sigma-Aldrich, Cat: 229857), and 704 ng/mL phosphorylethanolamine (Sigma-Aldrich, Cat: P0503). DTX-resistant (DR) PC3, DU145, and 22Rv1 cell lines were developed as described previously [101] and maintained in RPMI-1640 medium containing 10 nM DTX (LC Laboratories, Woburn, MA, USA; Cat: D-1000). LNCaP-ENZR cells were developed as previously described [102] and cultured in RPMI-1640 SGM supplemented with 10% FBS, 1% pen/strep, and Normocin 1G and maintained in medium containing 30 μM ENZ (MedChem Express, Monmouth Junction, NJ, USA; Cat: HY-70002). All cell lines were grown at 37 °C in a humidified incubator with 5% CO_2_. Mycoplasma contamination was routinely monitored at least twice per year using the Lonza MycoAlert^TM^ Mycoplasma Detection Kit (Lonza, Basel, Switzerland; Cat: LT07-218). Short tandem repeat (STR) profiling from ATCC (Cat: ATCC-135-XV) was utilized to authenticate the cell lines.

### 4.2. Antibodies

Antibodies specifically targeting ENO1 were acquired from Santa Cruz Biotechnology (mouse monoclonal L-27; Dallas, TX, USA; Cat: SC-100812) and Abcam (rabbit monoclonal EPR19758; Cambridge, UK; Cat: ab227978). Antibodies specifically targeting ENO2 (NSE) were acquired from Invitrogen (rabbit monoclonal 10H7L13; Waltham, MA USA; Cat: 702739) and Cell Signaling Technology (rabbit monoclonal E7D7I; Danvers, MA, USA; Cat: 65162S). Other primary antibodies included rabbit monoclonal anti-GAPDH 14C10 (Cell Signaling Technology; Cat: 2118S), rabbit polyclonal anti-LEDGF/p75 antibody A300-848 (Bethyl Laboratories/Fortis Life Sciences, Montgomery, TX, USA; Cat: A300-848A), rabbit polyclonal anti-CHGA antibody (Cell Signaling Technology; Cat: 60893S), rabbit monoclonal anti-Beta-Actin antibody D6A8 (Cell Signaling Technology; Cat: 12620S), rabbit monoclonal anti-p-H2A.X (Cell Signaling Technology; Cat:9718T), rabbit monoclonal anti-NCAM1 (CD56) (Cell Signaling Technology; Cat: 99746T), human anti-PARP (from the Casiano Lab de-identified human autoimmune serum collection), and mouse monoclonal anti-PSMA antibody 1H8H5 (Invitrogen, Waltham, MA, USA; Cat: 37-3900). Secondary fluorescence antibodies included goat anti-rabbit IgG StarBright^TM^ Blue 700 (Bio-Rad, Hercules, CA, USA; Cat: 12004161), goat anti-mouse IgG (H+L) Alexa Fluor^TM^ 488 (Invitrogen, Cat: A11001), goat anti-rabbit IgG (H+L) Alexa Fluor^TM^ 488 (Invitrogen, Cat: A11008), goat anti-human IgG DyLight^TM^ 680 (Invitrogen, Cat: SA5-10138). For chemiluminescent immunoblotting we used a horseradish peroxidase (HRP)-linked goat anti-rabbit antibody (Cell Signaling Technology; Cat: 7074S).

### 4.3. Immunoblotting

Whole-cell lysates were prepared for immunoblotting as previously described [71]. Briefly, total protein concentration in the lysates was quantified using the Bio-Rad DC Protein Assay Kit (Cat: 5000112) to ensure consistent loading of protein amounts across all lanes. Proteins were resolved using Mini-PROTEAN^®^ TGX^TM^ 4–15% precast gels (Bio-Rad, Cat: 4561084), followed by electrophoretic transfer onto nitrocellulose membranes (0.45 μm) (Bio-Rad, Cat: 1620167). Membranes were blocked using EveryBlot blocking buffer (Bio-Rad, Cat: 12010020) and incubated with specific primary antibodies, used at 1:2000 dilution, to ENO1, ENO2, GAPDH, PSMA, and CD56. Membranes were also blocked using 5% milk and incubated with antibodies to p-H2A.X (1:1000), LEDGF/p75 (1:2000), PARP (1:200), and CHGA (1:2000). After washing with tris-buffered saline with 0.02% Tween 20 (TBS-T), membranes were exposed to the appropriate secondary antibodies (1:5000). Following additional washes of membranes with TBS-T, immunoreactive bands were visualized either by fluorescence or chemiluminescence using the Bio-Rad ChemiDoc MP Imaging System. For the CHGA blots, a membrane previously used for PSMA immunoblotting was stripped by incubating in Restore PLUS Western Blot Stripping Buffer (Thermo Fisher Scientific, Waltham, MA, USA; Cat: 46430) for 10 min at room temperature with gentle agitation. This was followed by three washes of 5 min each in TBS-T 0.2% (*v*/*v*) before re-blocking and probing with CHGA. 

Bands corresponding to each target protein were quantified from a minimum of three independent experiments using either Bio-Rad Image Lab software (v6.1.0, build 7) or ImageJ software (National Institutes of Health, Bethesda, MD, USA, Fiji Version 1.44a), and relative protein expression was calculated by normalizing values to GAPDH expression. For Figure 1B, relative ENO1 or ENO2 protein expression from the immunoblotting analysis for each cell line and condition was determined by dividing the protein expression for each sample (Si) by the combined total protein expression of ENO1 or ENO2 (STotal ENO1 or STotal ENO2) within each DTX-sensitive and DTX-resistant cell line pair, resulting in values expressed as Si/STotal ENO1 or Si/STotal ENO2 for comparison. The brightness and contrast of immunoblotting images were adjusted to enhance band visualization while preserving the integrity of the original data. Images of the original immunoblots used in this study are shown in Supplementary Figures S12–S17.

### 4.4. Confocal Microscopy

For visualization of ENO1 and ENO2 on the cell surface, PCa cells were seeded directly onto glass coverslips, placed in 6-well plates, and allowed to adhere for 48 h. Cells were then washed twice with ice-cold PBS and incubated with anti-ENO1 antibody (L-27, 1:50) or anti-ENO2 antibody (10H7L13, 1:50) in PBS for 2 h at room temperature. Following incubation with primary antibodies, cells were washed and then incubated with either anti-mouse IgG (H+L) Alexa Fluor^TM^ 488 or anti-rabbit IgG (H+L) Alexa Fluor^TM^ 488 (1:100) for 45 min at room temperature in the dark. After washing, coverslips were mounted using VECTASHIELD^®^ Antifade Mounting Medium with DAPI (Vector Laboratories, Burlingame, CA, USA, Cat: H-1200-10). Confocal images were acquired using a Zeiss LSM 710 NLO microscope (Carl Zeiss, Oberkochen, Germany) equipped with a 63x oil immersion objective and appropriate filter sets. Image analysis was performed using ImageJ software Version 1.54g. The brightness and contrast of the confocal microscopy images were adjusted uniformly across each image to optimize visualization of fluorescent signals while maintaining the integrity of the original data.

### 4.5. Flow Cytometry 

A flow cytometric approach was used to quantify the total and surface abundance of ENO1 and ENO2 in PCa cells. To assess cell surface abundance, PCa cells cultured in HGM and SGM were incubated with either anti-ENO1 or anti-ENO2 specific antibodies (1:100, single stain) for 2 h at room temperature in the dark. Following incubation with primary antibodies, cells were then stained with secondary antibodies, either goat anti-mouse IgG (heavy plus light chains, H+L) Alexa Fluor^TM^ 488 or goat anti-rabbit IgG (H+L) Alexa Fluor^TM^ 488 (1:100), for 45 min at room temperature in the dark. Cells were then washed and fixed with 1% paraformaldehyde. To assess total ENO1 and ENO2 protein abundance, cells were permeabilized with 90% methanol diluted in PBS for 5 min at 4 °C prior to incubation with anti-ENO1 or anti-ENO2 primary antibodies for 2 h at room temperature in the dark, followed by incubation with the appropriate Alexa Fluor^TM^ 488 secondary antibodies. Flow cytometric analysis was performed using a MACSQuant Analyzer 10 (Miltenyi Biotec, Bergisch Gladbach, Germany), and data was processed with FlowJo software version 10 (FlowJo LLC, Ashland, OR, USA). Cells were first gated based on forward- and side-scatter characteristics to identify the main intact-cell population and exclude debris, followed by singlet discrimination. The same gating and analysis workflow was applied to all cell lines used in the study. Secondary-only controls were included for each cell line and staining condition to determine background fluorescence. Raw median fluorescence intensity (MFI) values were calculated for antibody-stained samples and their matched secondary-only controls. Background corrected MFI was calculated by subtracting the MFI of its matched secondary antibody-only control from the raw MFI of the antibody-stained sample. For chart visualization of flow cytometry data, ENO1 or ENO2 relative fluorescence intensity (RFI) values for each condition were normalized to the combined signal intensity of the two proteins. This was accomplished by dividing the RFI for each sample (Si) by the combined RFI of ENO1 and ENO2 (STotal ENO1 + ENO2) within each matched DTX-sensitive compared to DTX-resistant cell line (Figure 3A–H), LASCPC-01 compared to NCI-H660 (Figure 3I–L, Supplementary Figure S6), and LNCaP compared to LNCaP-ENZR (Figure 3M–P). Resulting normalized values are reported as Si/STotal ENO1 + ENO2 for comparative analyses. Normalization to individual ENO1 or ENO2 protein abundance was performed by dividing the RFI for each sample (Si) by the total RFI of either ENO1 or ENO2 (STotal ENO1 or STotal ENO2) again within each DTX-sensitive vs DTX-resistant cell line (Supplementary Figure S4). These values are reported as Si/STotal ENO1 or Si/STotal ENO2 for comparison. For the LASCPC-01, NCI-H660, LNCaP, and LNCaP-ENZR cell lines, ENO values were calculated for inter-cell line (Figure 3I,J,M,N; Supplementary Figure S7C,D) or per-cell line (Figure 3K,L,O,P; Supplementary Figure S7A,B), with comparisons following the same normalization procedures outlined above (Si/STotal ENO1 + ENO2). 

For cell-cycle analysis, DTX-resistant cell lines were seeded in 6-well plates at 1 × 10^5^ cells per well and allowed to adhere for 24 h. Cells were then treated with ENO1 mAb (L-27) concentrations approximating their respective IC_50_ and IC_75_ (PC3-DR: 1.5 and 1.9 µg/mL; DU145-DR: 3.7 and 4.7 µg/mL, respectively), determined by nonlinear dose–response curve analysis. Mouse IgG isotype control was applied to each cell line at the IC_75_ equivalent concentration Treatments were performed for 72 h in at least three independent experiments. After treatment, cells and supernatants were collected, pooled, centrifuged at 500× *g* for 5 min at 4 °C, washed with PBS, and fixed by slow addition of 2 mL ice-cold 70% ethanol while gently vortexing, then stored at −20 °C for at least 1 h. Ethanol-fixed cells were then centrifuged, washed in PBS, resuspended in 0.5 mL FxCycle^TM^ PI/RNase Staining Solution (Thermo Fisher Scientific, Cat: F10797), incubated for 30 min at room temperature in the dark, and analyzed on a MACSQuant10 flow cytometer (Miltenyi Biotec) using the PI/PerCP channel. Cell-cycle phase distributions (G_0_/G_1_, S, G_2_/M) were obtained from PI histograms after doublet discrimination using FlowJo software version 10 (FlowJo LLC). 

For analysis of cell death, cells were seeded in 6-well plates at a density of 1 × 10^5^ cells per well 24 h prior to ENO1 mAb (L-27) treatment and maintained at 37 °C in a humidified atmosphere of 5% CO_2_. Cells were then treated with ENO1 mAb concentrations approximating their respective IC_50_ and IC_75_, or mouse IgG isotype control, as described above. Treatments were performed for 72 h in at least three independent experiments. Cells were then detached using mild trypsinization and samples were transferred to a 96-well plate at 1.5 × 10^5^ cells per well for staining. Cells were double-stained using the Pacific Blue^TM^ Annexin V Apoptosis Detection Kit with 7-AAD (BioLegend, San Diego, CA, USA; Cat: 640926) to distinguish early apoptotic, late apoptotic/secondary necrotic, and viable cell populations. Apoptotic cell quantification was performed by flow cytometry using a MACSQuant^®^ Analyzer 10 (Miltenyi Biotec), and data were analyzed using FlowJo software version 10 (FlowJo LLC). Intact cells were first gated based on forward and side scatter to exclude cellular debris. Annexin V and 7-AAD signals were visualized using density plots, and apoptotic and necrotic populations were defined by quadrant analysis.

### 4.6. RNA Interference

PC3-DR and DU145-DR cells (75,000 cells per well) were cultured on 6-well plates and transfected after 24 h with either scrambled (SD) negative control (Integrated DNA Technologies, Coralville, IA, USA; Cat: 51-01-19-09) or combination of 12.5 nM hs.Ri.ENO1.13.1 and hs.Ri.ENO1.13.3 duplex siRNA oligos for up to 96 h. Transfections were performed using Interferin^®^ siRNA transfection reagent (Polyplus-transfection^®^, Illkirch, France; Cat: 409-01) as described previously [102] and confirmed by immunoblotting. The duplex siRNA sequences used were as follows:

hs.Ri.ENO1.13.1

5′-UACACUGAUAAGGUGGUCAUCGGCA-3′

3′-CGAUGUGACUAUUCCACCAGUAGCCGU-5′

hs.Ri.ENO1.13.3

5′-GUCAUUGUUUUUCUCGCCUCACUTT-3′

3′-UUCAGUAACAAAAAGAGCGGAGUGAAA-5′

### 4.7. RNA Extraction and Real-Time Quantitative PCR

After ENO1 depletion, total RNA was extracted from cultured cells using TRI Reagent^®^ solution (Molecular Research Center, Cincinnati, OH, USA; Cat# TR 118). The iScript cDNA synthesis kit (Bio-Rad, Cat# 1708891) was used to reverse transcribe RNA (0.8 μg) into cDNA. Primer sequences for ENO1, ENO2 and GAPDH were synthesized by Integrative DNA Technologies. Quantitative polymerase chain reaction (qPCR) was performed on BioRad’s CFX96 Touch Real-Time PCR detection system using iSYBR Green Supermix (Bio-Rad, Cat# 1708880). Thermal cycling conditions were 95 °C for 2 min, 95 °C for 5 s, and 60 °C for 30 s for 39 cycles, followed by melt analysis from 65 to 95 °C. Expression levels were normalized to GAPDH. Experiments were performed with at least four independent biological replicates, each analyzed in technical triplicates. Relative expression was normalized to the corresponding control. Primers used for qPCR were:

ENO1

5′-AGTCAACCAGATTGGCTCCGTG-3′

3′-GAAGTAGCGACTGGACCAACAC-5′

ENO2

5′-CTGTATCGCCACATTGCTCAGC-3′

3′-CGAGAGTACGACCGTTGTTCGA-5′

GAPDH

5′-CGAGATCCCTCCAAAATCAA-3′

3′-TACAAGCAGTACCCACACTT-5′

### 4.8. Cell Proliferation Assays

Cells were seeded in 96-well plates at a density of 3200 cells per well, allowed to adhere for 24 h, and then treated with increasing concentrations of DTX (1, 5, 10, 50, 100 and 500 nM) for up to 72 h in at least three independent experiments, each performed with three biological replicates. Cell proliferation was measured using the MTT Cell Proliferation Assay (ATCC, Cat:30-1010K). Briefly, after treatments, 3-(4,5-dimethylthiazol-2-yl)-2,5-diphenyltetrazolium bromide (MTT) was added to each well (5 mg/mL), and plates were incubated for 2 h in a 5% CO2 incubator at 37 °C. Plates were then centrifuged at 500× *g* for 5 min to avoid loss of floating cells. Supernatants were discarded, and 100 μL of dimethyl sulfoxide (DMSO, Thermo Fisher Scientific; Cat: D128-1) was added to each well. Absorbance was measured at 570 nm using a SpectraMax spectrophotometer (Molecular Devices LLC, San Jose CA, USA). Values were normalized to the absorbance obtained for untreated, control cells. Standard deviation (SD) was calculated, and IC_50_ values were extrapolated using GraphPad Prism version 10 (GraphPad Software, San Diego, CA, USA).

For crystal violet assays, cells were seeded in 96-well plates at a density of 4000 cells per well, allowed to adhere for 24 h, and then treated with increasing concentrations of ENO1 mAb (L-27; 1, 5, 10, and 15 μg/mL) or mouse IgG isotype control (15 μg/mL, Cell Signaling Technology; Cat: 68860L) for 72 h in at least three independent experiments, each performed with three biological replicates. After the 72 h incubation, cells were washed once with PBS and fixed with 4% paraformaldehyde (*v*/*v* in PBS) for 15–30 min at room temperature. Plates were then washed with PBS, residual buffer was removed, and 0.1% (*w*/*v*) crystal violet in water was added to each well (100 μL/well) and incubated for 30 min at room temperature. Plates were rinsed thoroughly in water until background staining was minimal and allowed to dry completely on the bench. Bound dye was solubilized by adding 100 μL of 10% acetic acid (*v*/*v* in water) to each well. Absorbance was measured at 570 nm using a SpectraMax microplate spectrophotometer (Molecular Devices LLC, San Jose, CA, USA) with SoftMax Pro software version 6.5.1. Values were normalized to the absorbance obtained for IgG isotype control wells. Standard deviation and IC_50_ values for ENO1 antibody were determined using GraphPad Prism version 10 (GraphPad Software, San Diego, CA, USA). 

### 4.9. Clonogenic Assays

Cells were transfected with individual siRNAs and cultured in RPMI-1640 medium (SGM or HGM) supplemented with 10% FBS for 72 h. Post-transfection, cells were seeded into 6-well plates at a density of 3600 cells per well in complete growth medium and allowed to adhere for 48 h at 37 °C in a humidified incubator with 5% CO_2_. For clonogenic assays using DTX treatment, the medium was then replaced with fresh medium containing DTX at concentrations of 10, 20, 40, 60, or 100 nM, and cells were incubated for 10 days to allow colony formation. For clonogenic assays using ENO1 mAb treatment, non-transfected DTX-resistant cells were seeded and allowed to adhere as described above, after which the medium was replaced with fresh complete medium containing ENO1 mAb at 1.0, 1.5, or 2.0 µg/mL, or IgG isotype control at 2.0 µg/mL. Cells were incubated for 10 days to allow colony formation. At the end of the incubation period, wells were gently washed with PBS and fixed with a 1:3 acetone:methanol (*v*/*v*) solution for 5 min. Fixed cells were stained with 0.5% (*w*/*v*) crystal violet for 20 min at room temperature. Excess stain was removed by rinsing with distilled water and plates were air-dried. Images of stained colonies were acquired using the Bio-Rad ChemiDoc MP Imaging System, and colony formation was quantified by measuring the total area covered by colonies using ImageJ software with the ColonyArea plug-in [103].

### 4.10. Tumorsphere Formation Assay

NCI-H660 (30,000 cells/well) and LASCPC-01 (2000 cells/well) cells were seeded into 24-well ultra-low-attachment plates (Genesee Scientific; Cat: 25-102) at 0.5 mL per well in MammoCult^TM^ medium (Stemcell Technologies, Vancouver, BC, Canada; Cat: 05620), supplemented with hydrocortisone (0.48 μg/mL; Sigma-Aldrich, St. Louis, MO, USA; Cat: H0135), heparin (4 μg/mL; Sigma-Aldrich; Cat: H3149), and 1% penicillin/streptomycin. The MammoCult^TM^ medium contained ENO1 (L-27) mouse mAb at final concentrations of 1.5 or 2.0 μg/mL, or mouse IgG isotype control at 2.0 μg/mL. Tumorspheres were grown for 7 days at 37 °C in a humidified incubator with 5% CO_2_ and visualized using an Olympus IX70 microscope equipped with phase-contrast and HMC optics. Images of tumorspheres were acquired using the SPOT imaging system, and tumorsphere area was quantified from at least three independent images per treatment condition using ImageJ software.

### 4.11. Analysis of Gene Expression in Clinical Prostate Tumors

Publicly available transcriptomic and clinical data for TCGA-PRAD cohortwere retrieved from the National Cancer Institute Genomic Data Commons using the TCGAbiolinks R package. GDC STAR count data were processed in R (version 4.5.2) to obtain TPM-normalized expression values for ENO1, ENO2, and MYC. Samples were classified by TCGA barcode as primary tumor (sample type 01) or normal tissue (sample type 11). Tumors were further grouped by Gleason score (≤6, 7, 8, or ≥9). In addition, publicly available transcriptomic and clinical data for the SU2C/PCF metastatic PCa cohort were obtained from cBioPortal using the cBioPortalData R package (version 2.22.1). Poly(A)-selected RNA-seq FPKM values from 266 samples were converted to TPM using all genes detected in each sample. ENO1 and ENO2 expression values were matched to pathological classifications. Samples classified as “Adenocarcinoma” formed the adenocarcinoma group; those classified as “Adenocarcinoma with NE features” or “Small cell” were combined into the “Adeno/NE Features + Small Cell” group. Samples with inadequate or missing pathological classifications were excluded. For patients with multiple samples, expression values for each gene were averaged within patient before analysis. Expression data were displayed as violin plots with lines indicating the median and interquartile range. 

### 4.12. Statistical Analyses

Statistical analyses were performed using GraphPad Prism version 10 unless otherwise specified. Two-sample comparisons were analyzed using the two-tailed Student’s *t*-test. Multiple comparisons were performed using one-way or two-way ANOVA, as appropriate. Data are presented as mean ± SEM from at least three independent experiments. For TCGA-PRAD transcriptomic analyses, differences in ENO1, ENO2, and MYC transcript expression between “PRAD” Primary Tissue and “Normal” Solid Tissue were evaluated using the nonparametric Mann–Whitney U test in GraphPad Prism. Monotonic trends across increasing Gleason score were evaluated using the Jonckheere-Terpstra trend test in R (version 4.5.1). For SU2C/PCF transcriptomic analyses, ENO1 and ENO2 expression were compared within each pathology group using the Wilcoxon matched-pairs signed-rank test. A *p* value < 0.05 was considered statistically significant.

## Data Availability

The datasets used and analyzed in the current study are available from the corresponding author upon request. Requests for reagents or specific data may involve a Materials Transfer Agreement (MTA) and will be honored once the MTA process has been finalized by Loma Linda University Health and the institutions that request the data or resources.

## Supplementary Materials

The following supporting information can be downloaded at: https://www.mdpi.com/article/10.3390/ijms27219714/s1.
