# Supplementary material for: ENO1 Is More Abundant than ENO2 on the Surface of Metastatic and Neuroendocrine Prostate Cancer Cells and Upregulated in High-Risk Prostate Tumors
Source: Int J Mol Sci. 2026 Oct 29;27(21):9714. doi: 10.3390/ijms27219714 (PMC13762735; doi:10.3390/ijms27219714)
Supplement: Supplementary file 1 [file ijms-27-09714-s001.zip › ijms-4490645-supplementary.pdf]

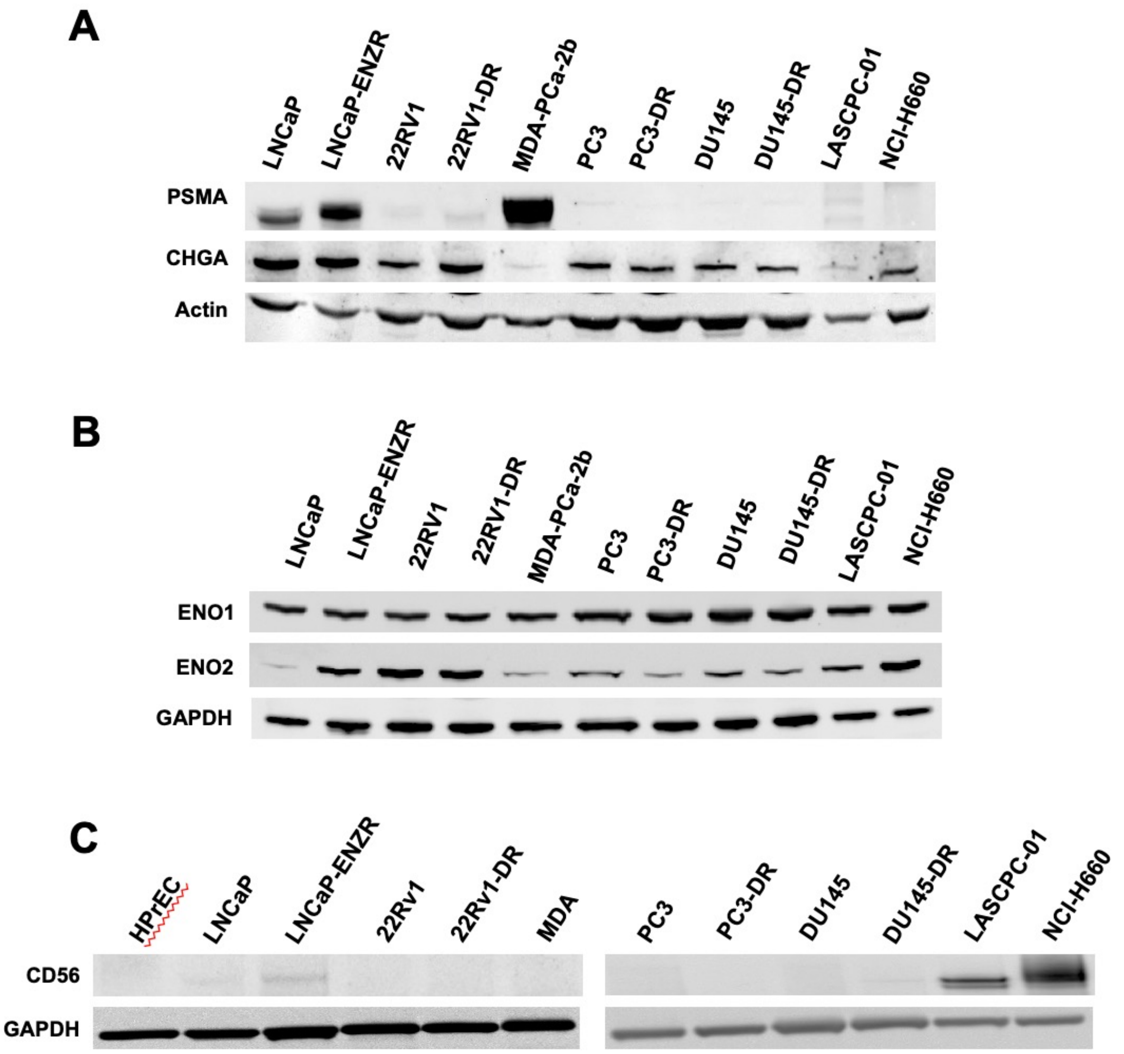

Supplementary Figure S1. PSMA is absent in mCRPC and NEPC cell lines. Immunoblots showing the protein expression profiles of PSMA, CHGA, ENO1, ENO2, and CD56 across a broad panel of PCa cell lines. β-actin and GAPDH were used as loading controls.

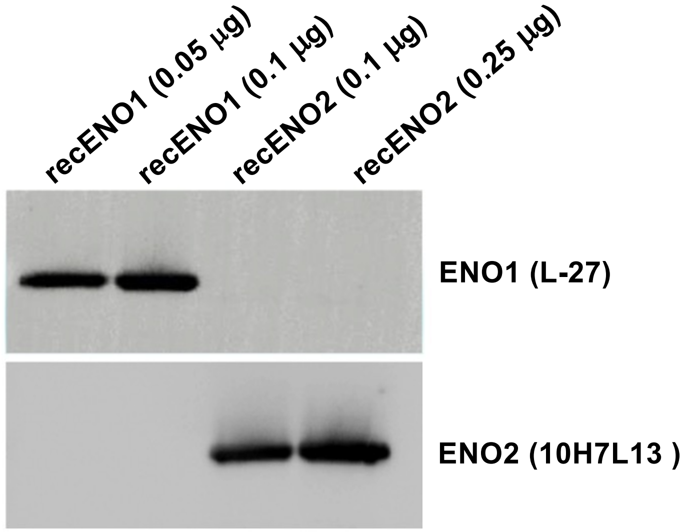

Supplementary Figure S2. Specificity of ENO1 and ENO2 antibody reactivity. Immunoblots showing the reactivity of anti-ENO1 (L-27) and anti-ENO2 (10H7L13) antibodies (1:2000 dilution) against recombinant ENO1 and ENO2 proteins.

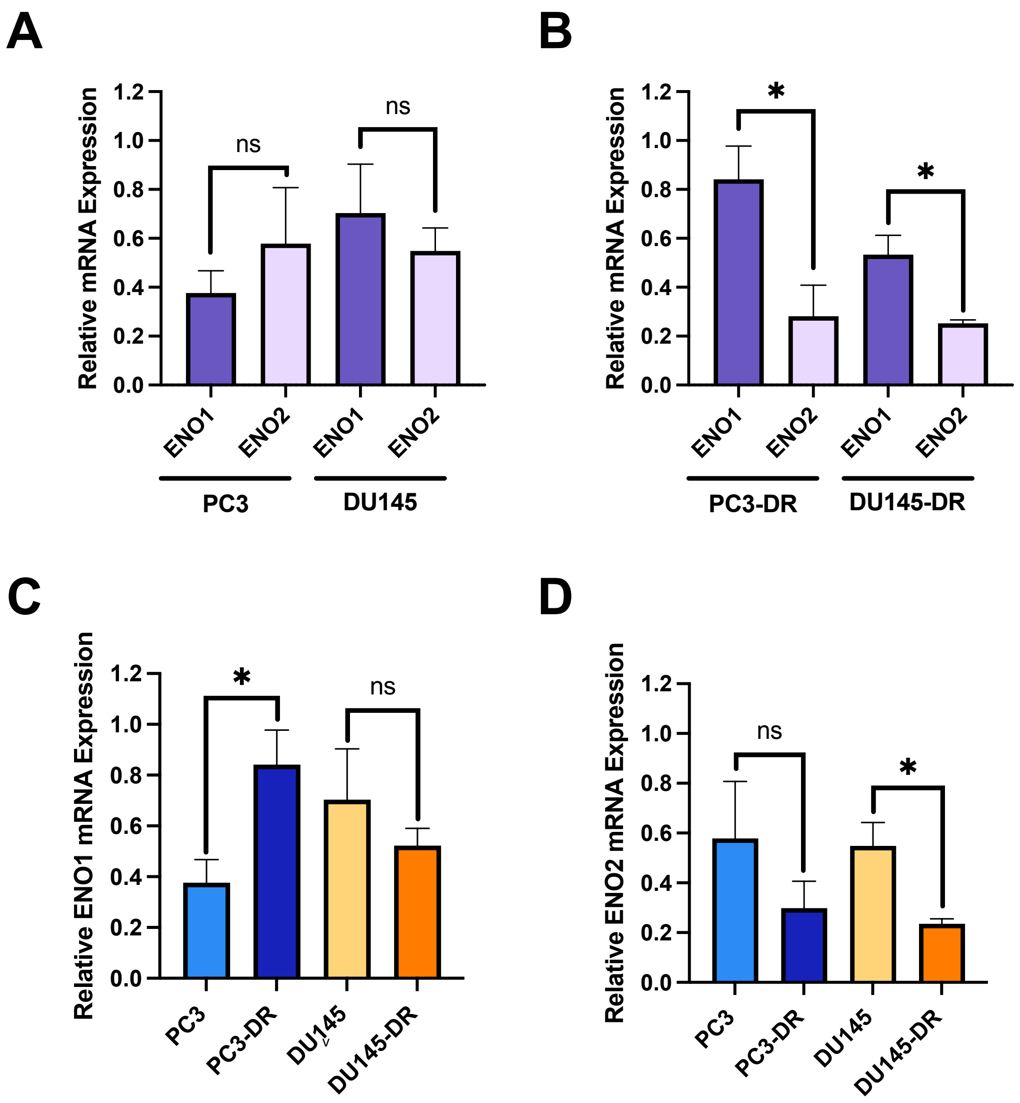

Supplementary Figure S3. ENO1 and ENO2 transcript expression in mCRPC cell lines. Real time-polymerase chain reaction (RT-PCR) analysis of relative transcript expression of ENO1 compared to ENO2 in **(A)** PC3 and DU145 cells and **(B)** PC3-DR and DU145-DR cells. RT-PCR analysis of relative expression of ENO1 **(C)** and ENO2 **(D)** in mCRPC DTX-sensitive and DTX–resistant cells.

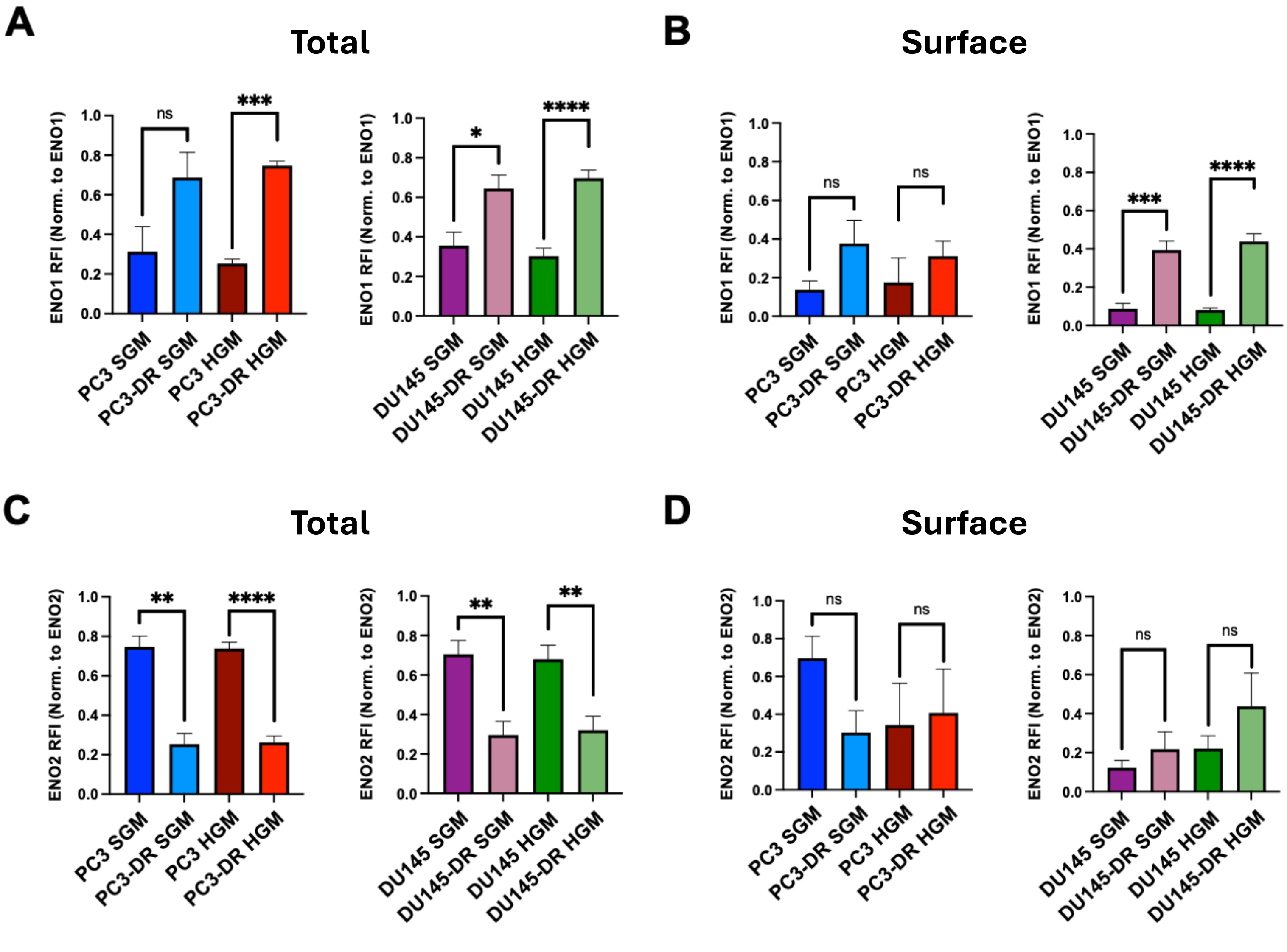

Supplementary Figure S4. Total and surface abundance of ENO1 and ENO2 in mCRPC cells. Flow cytometric quantification of normalized RFI values for **(A,C)** total and **(B,D)** surface abundance of ENO1 (antibody L-27) and ENO2 (antibody 10H7L13), respectively, in PC3, PC3-DR, DU145, and DU145-DR cells cultured in SGM and HGM. Data are presented as mean ± SEM. Statistical significance was determined using a two-tailed Student's *t*-test. * $p < 0.05$, ** $p < 0.01$, *** $p < 0.001$, **** $p < 0.0001$; ns = non-significant.

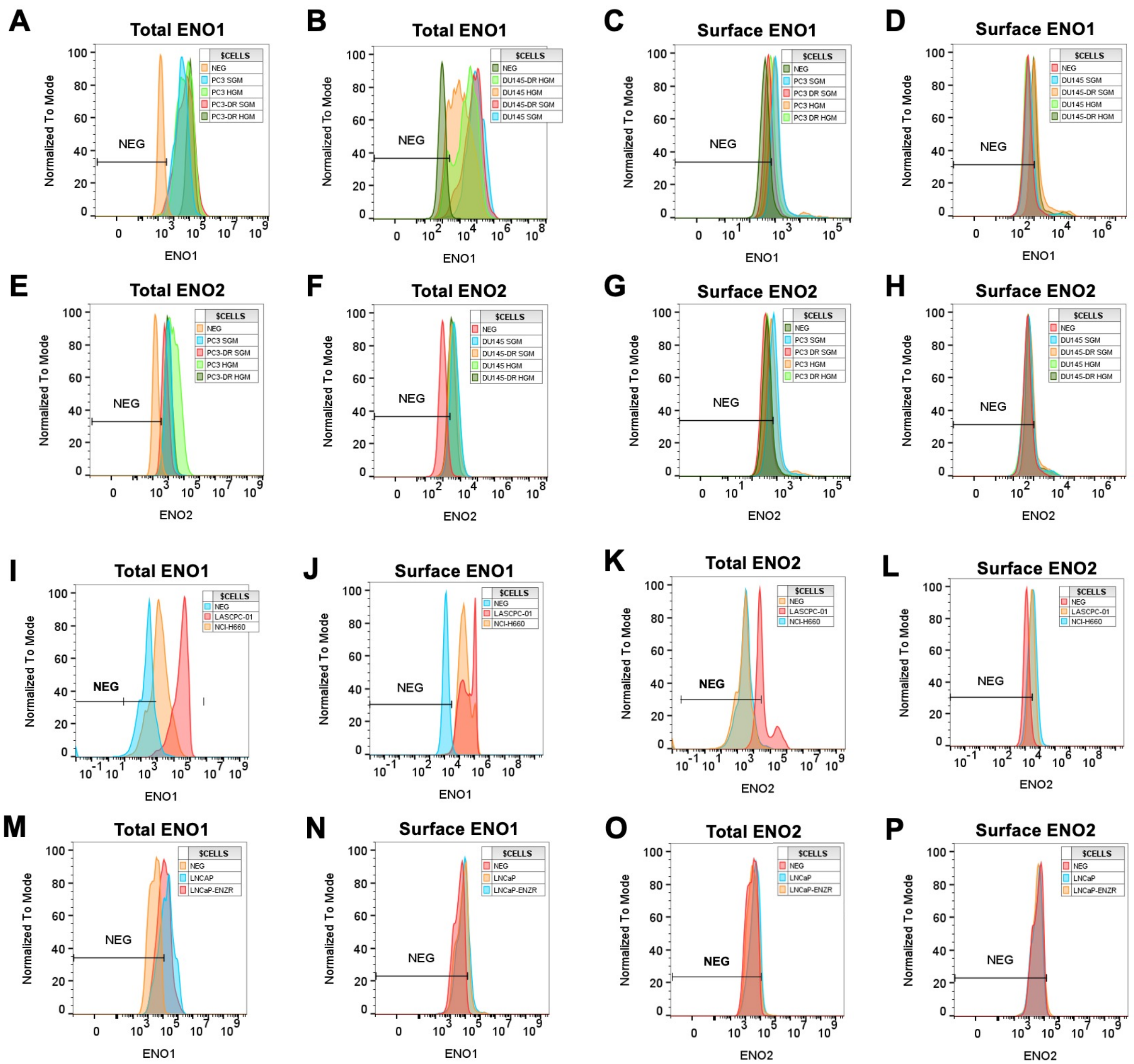

Supplementary Figure S5. Flow cytometry histograms reveal high total and surface abundance of ENO1. Representative flow cytometry histograms illustrate: **(A,B)** Total expression of ENO1 (antibody L-27) in PC3, PC3-DR, DU145 and DU145-DR cells cultured in SGM and HGM; **(C,D)** surface abundance of ENO1 in PC3, PC3-DR, DU145 and DU145-DR cells cultured in SGM and HGM; **(E,F)** total expression and **(G,H)** surface abundance of ENO2 (antibody 10H7L13) in the same cell lines; total expression **(I,K)** and surface abundance **(J, L)** of ENO1 and ENO2, respectively, in the NEPC cell lines LASCPC-01 and NCI-H660; and total expression **(M,O)** and surface abundance **(N, P)** of ENO1 and ENO2, respectively, in LNCaP and LNCaP-ENZR cell lines.

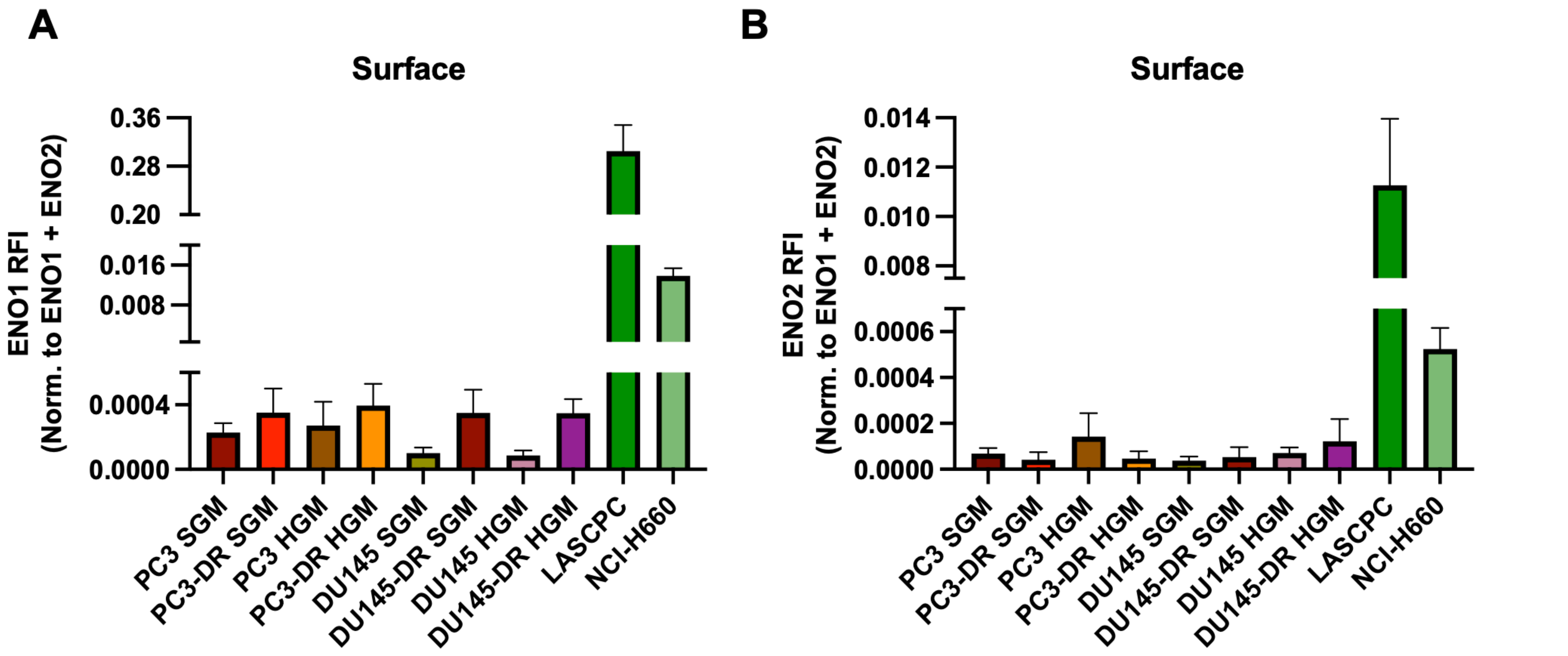

Supplementary Figure S6. ENO1 is more abundantly expressed on the surface of mCRPC and NEPC cells compared to ENO2. Relative surface abundance of **(A)** ENO1 (antibody L-27) and **(B)** ENO2 (antibody 10H7L13) in mCRPC cell lines (PC3, PC3-DR, DU145, DU145-DR) cultured under SGM or HGM conditions, and in NEPC cell lines (LASCPC-01 and NCI-H660).

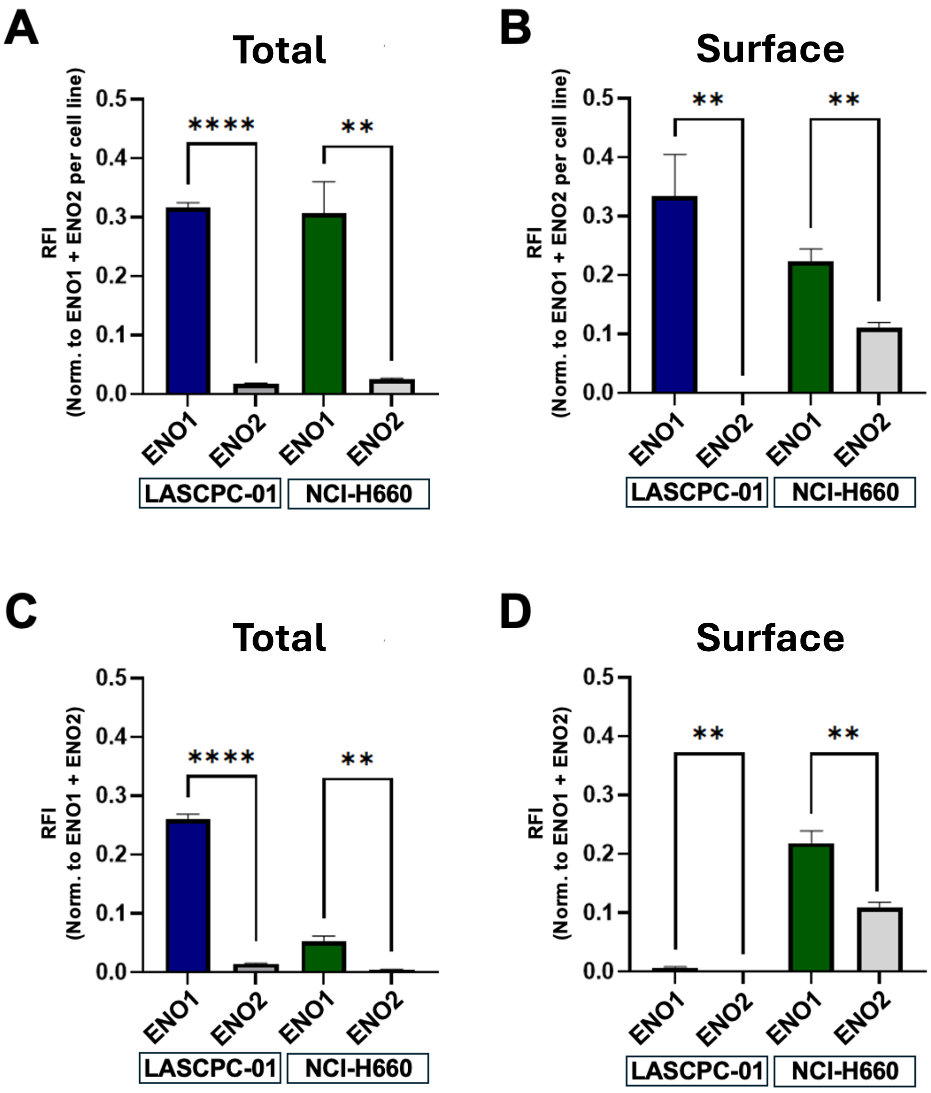

Supplementary Figure S7. Validation of ENO1 and ENO2 total expression and surface abundance using an alternative set of antibodies confirms elevated ENO1 levels relative to ENO2. (A) Total and **(B)** surface individual RFI values for ENO1 (antibody EPR19758) and ENO2 (antibody E7D7I) in LASCPC-01 and NCI-H660 cells are shown relative to each respective cell line (normalized to ENO1 + ENO2 per cell line). **(C)** Total and **(D)** surface RFI values for ENO1 and ENO2 in LASCPC-01 and NCI-H660 cells (normalized to ENO1 + ENO2). Data are presented as mean ± SEM. Statistical significance was determined using a two-tailed Student's t-test. ** $p < 0.01$, **** $p < 0.0001$.

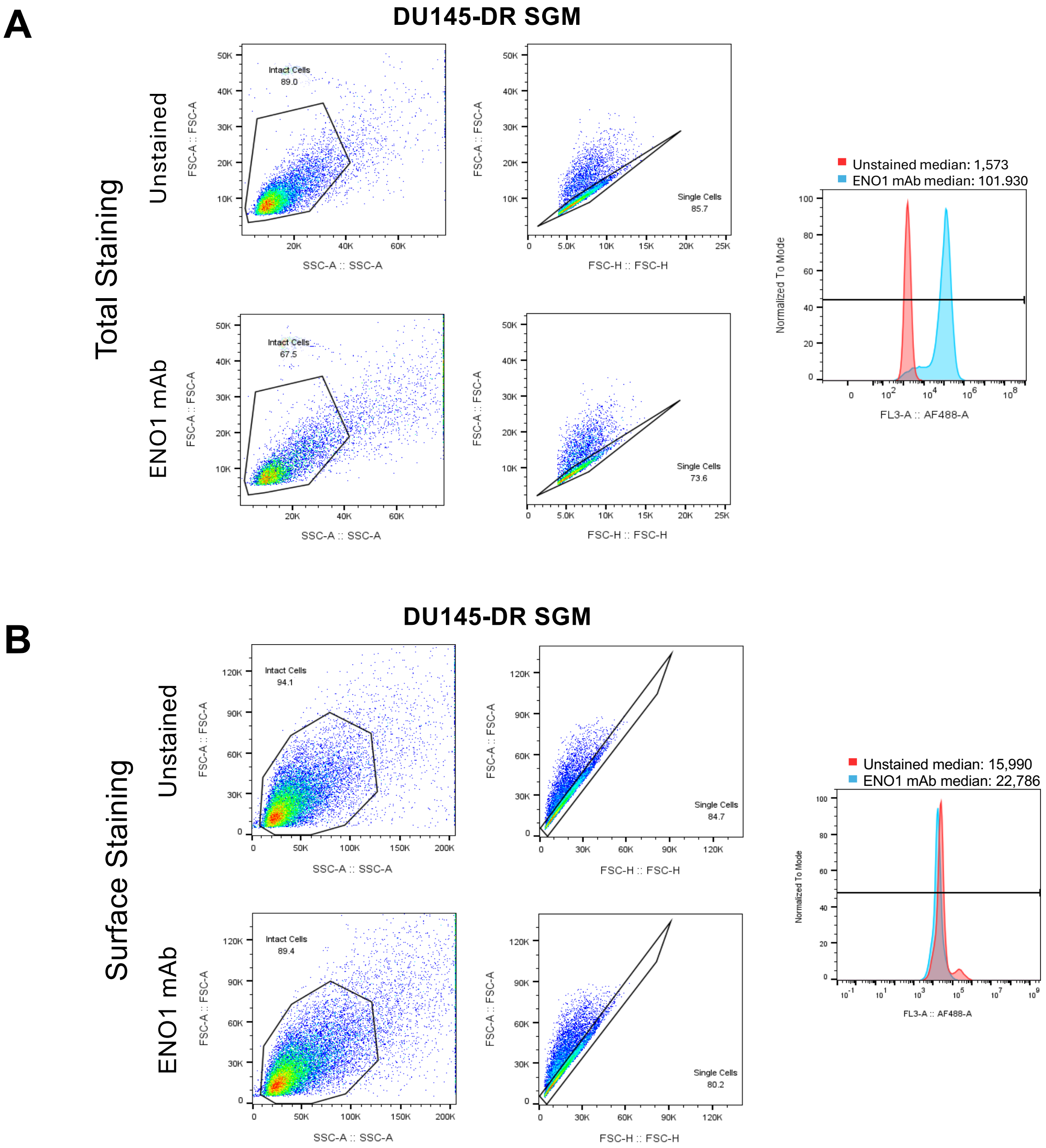

Supplementary Figure S8. Representative flow cytometry gating strategy for analysis of total and surface abundance of ENO1 and ENO2. (A) Representative gating of intact cells and singlets for total staining. **(B)** Representative gating of intact cells and singlets for surface staining. Plots show unstained and ENO1 antibody-stained DU145-DR cells as an example of the gating strategies used to measure ENO1 and ENO2 median fluorescence intensity (MFI) in various PCa cell lines.

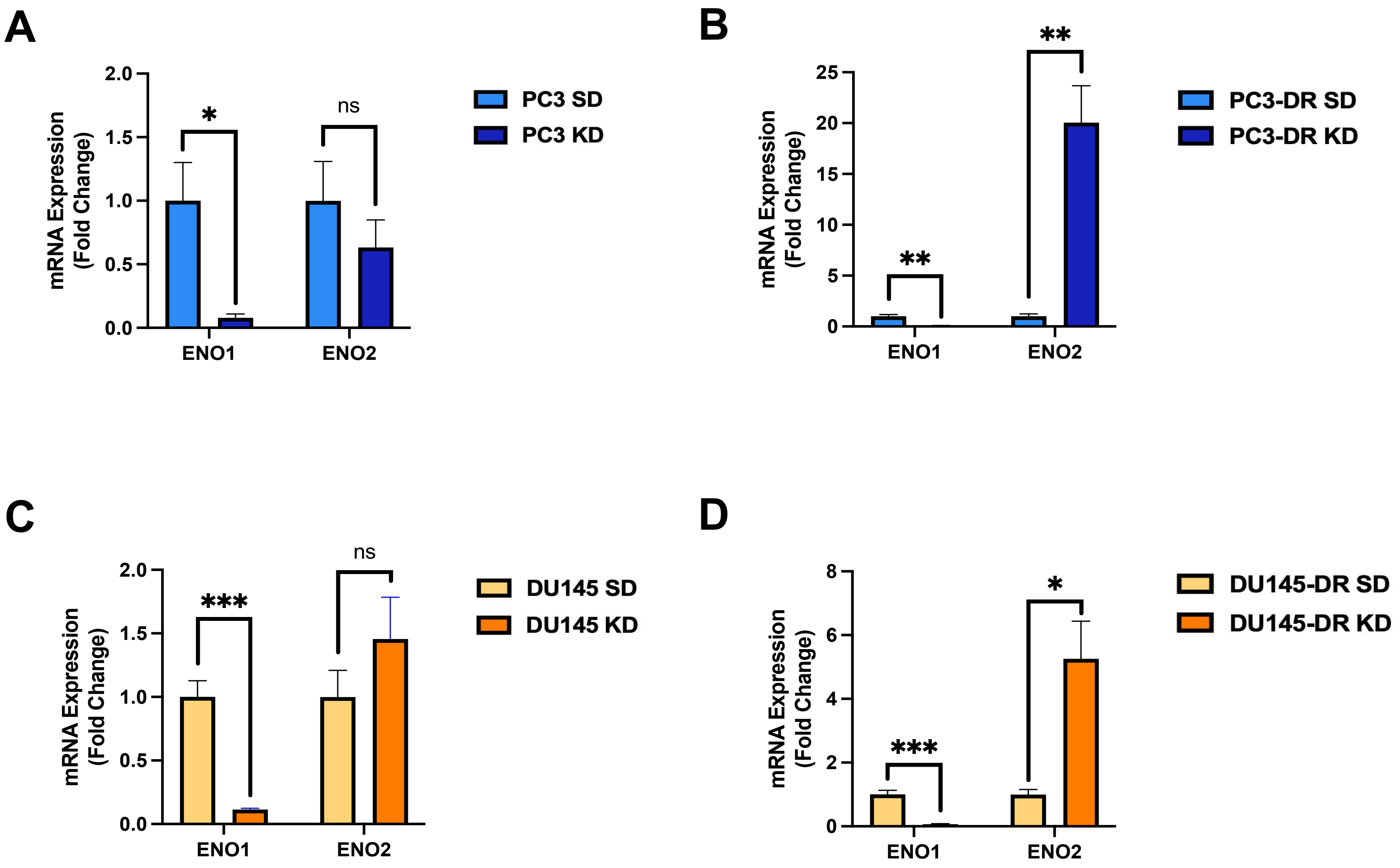

Supplementary Figure S9. ENO1 and ENO2 mRNA expression in mCRPC cell lines after ENO1 depletion. RT-PCR analysis of ENO1 vs ENO2 transcript expression fold change in PC3 **(A),** PC3-DR **(B),** DU145 **(C)** and DU145-DR **(D)** cells with scrambled duplex control oligos (SD) or ENO1 knockdown (KD).

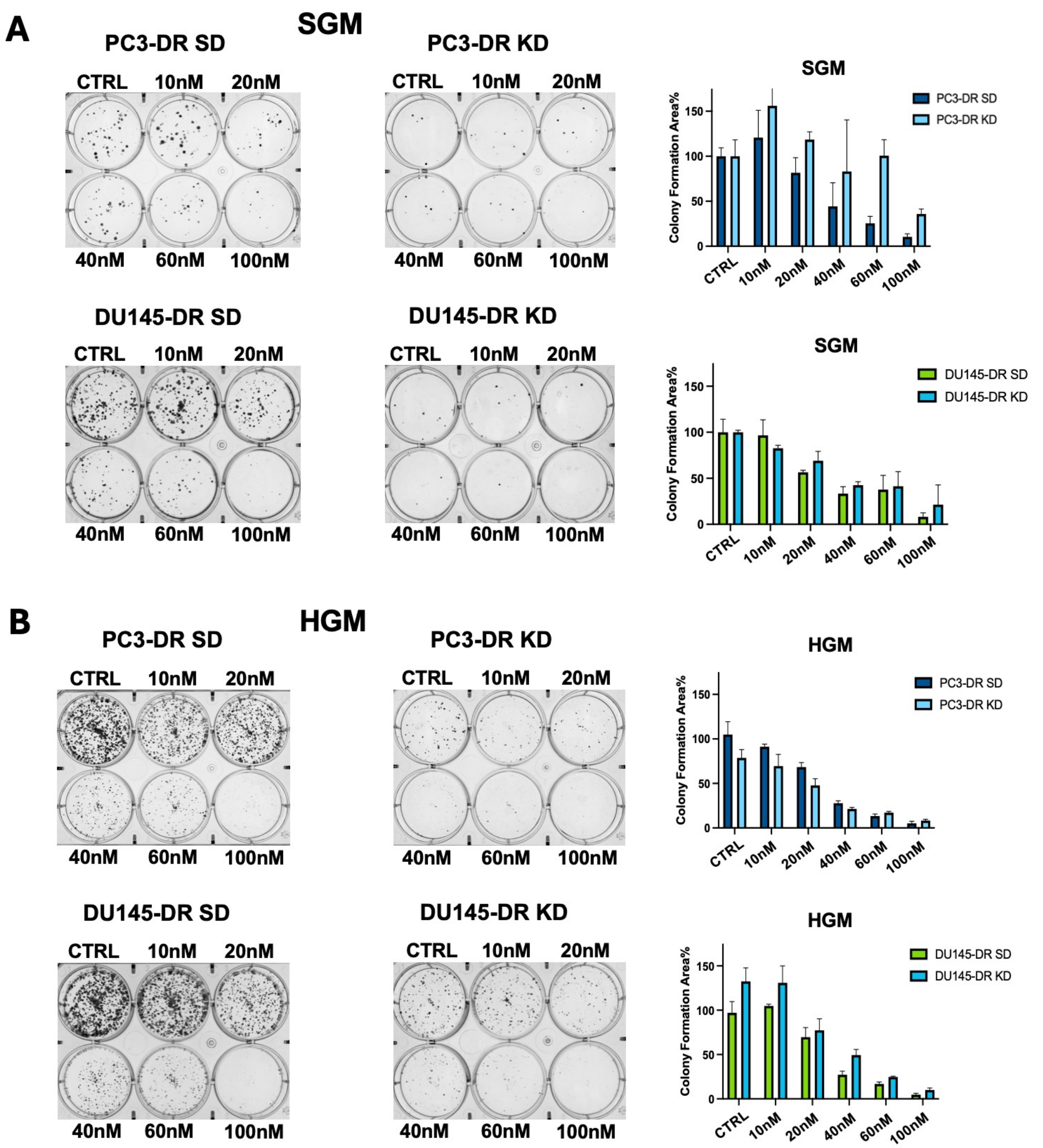

Supplementary Figure S10. ENO1 knockdown does not impact DTX sensitivity in DTX-resistant mCRPC cells. ENO1-depleted cells treated with increasing concentrations of DTX (10, 20, 40, 60, and 100 nM) do not exhibit resensitization to DTX in colony formation assays. Colony formation area percentage of ENO1 knockdown (KD) was normalized to SD controls in DTX-resistant PC3 and DU145 cells cultured in SGM **(A)** and HGM **(B)**. Colonies were analyzed 14 days post-seeding for SGM and 10 days post-seeding for HGM. Data are presented as mean ± SEM, normalized to the corresponding SD control.

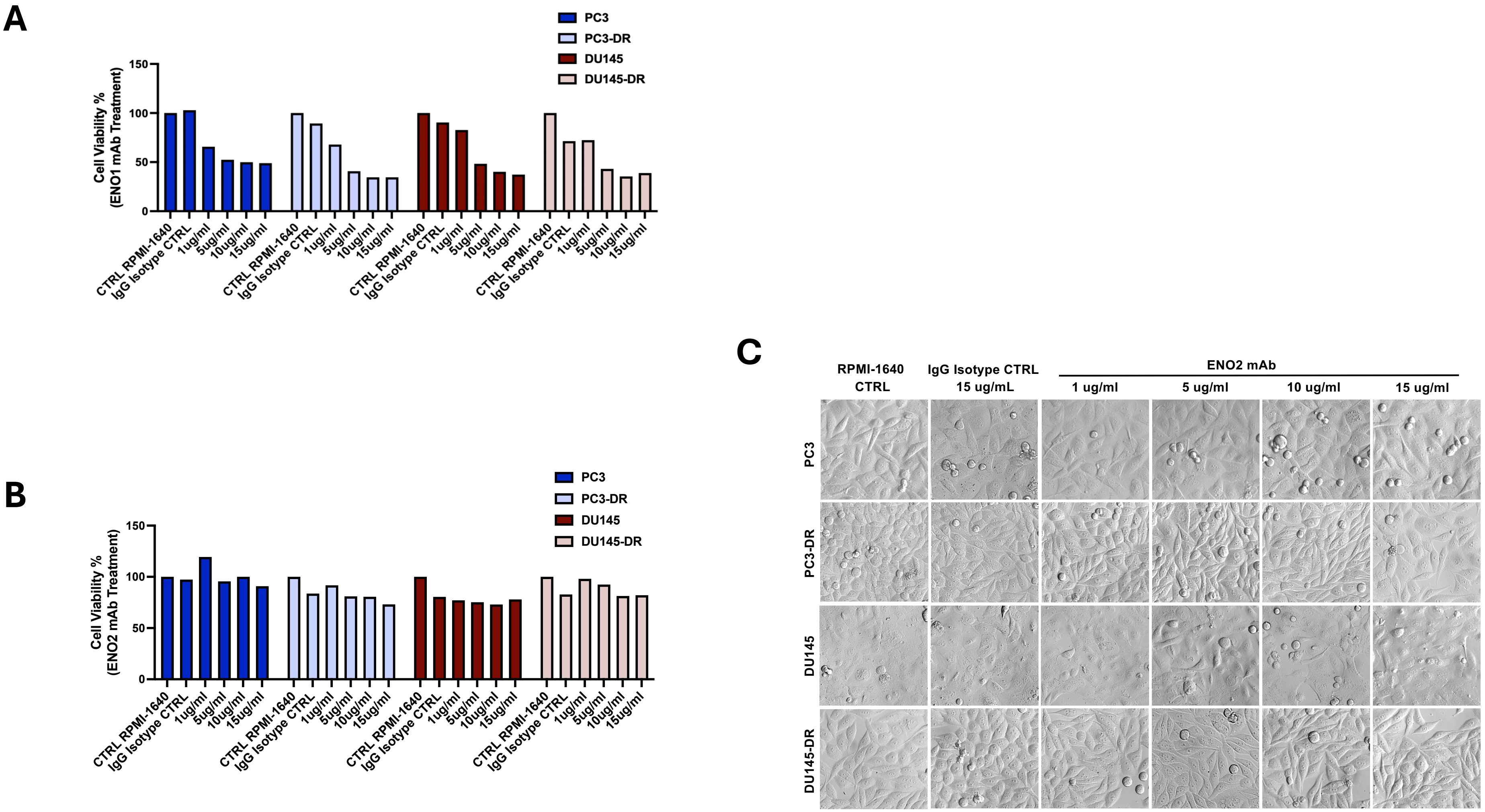

Supplementary Figure S11. Effects of ENO1 and ENO2 mAb treatment on DTX-sensitive and -resistant mCRPC cells. **(A–B**) Crystal violet assays showing cell viability in PC3, PC3-DR, DU145, and DU145-DR cells following treatment with the indicated concentrations of ENO1 mAb **(A)** or ENO2 mAb **(B)**. Viability is shown relative to the RPMI-1640 control; an IgG isotype control was also included. **(C)** Representative HMC microscopy images of cells treated with RPMI-1640, IgG isotype control, or the indicated concentrations of ENO2 mAb.

Supplementary Figure S12: Original Western blot images for Figure 1A

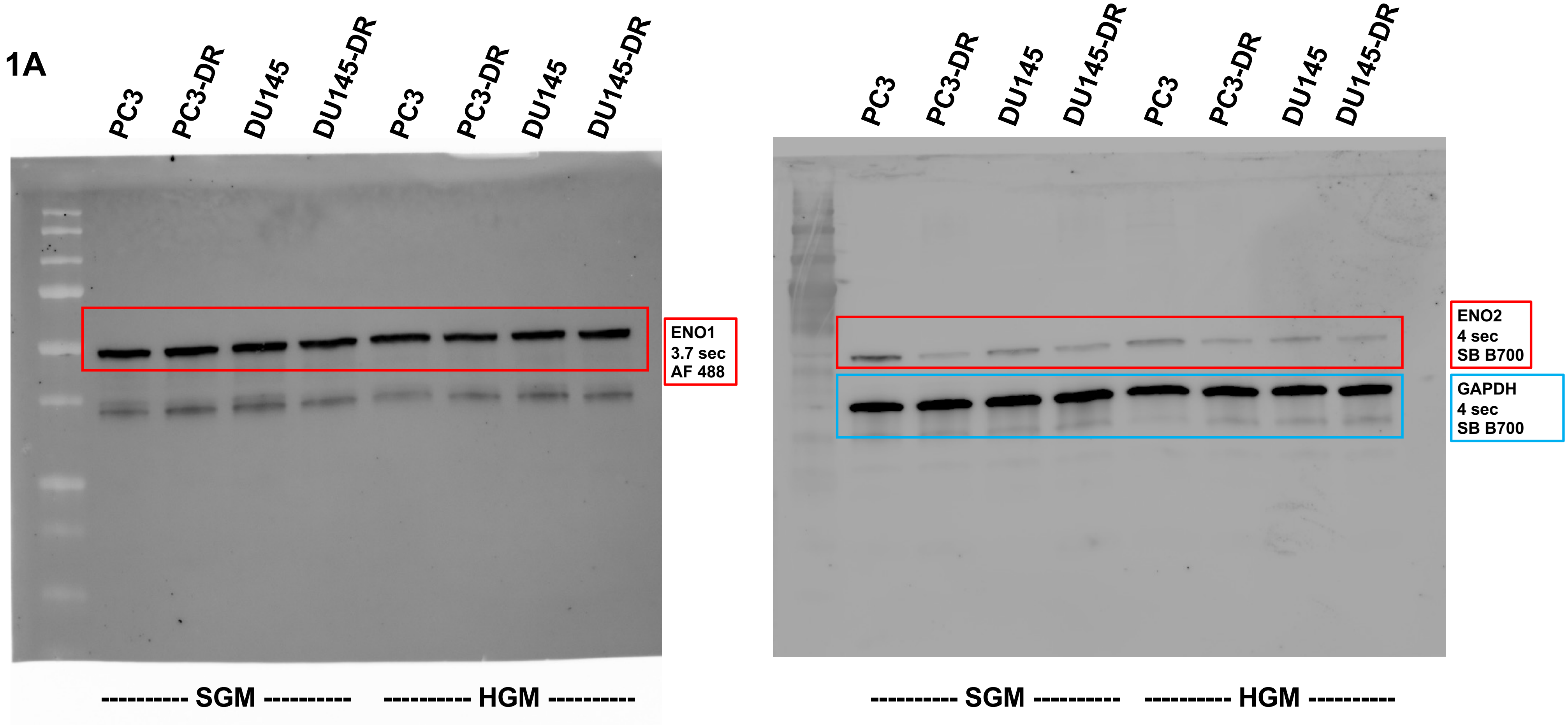

ENO1 and ENO2, which have similar molecular weights, were probed on the same membrane using different specific primary antibodies and corresponding fluorescently labeled secondary antibodies. Wavelengths were detected in a BioRad ChemiDoc Imaging System using different filters.

Supplementary Figure S13: Original Western blot images for Supplementary Figure S2

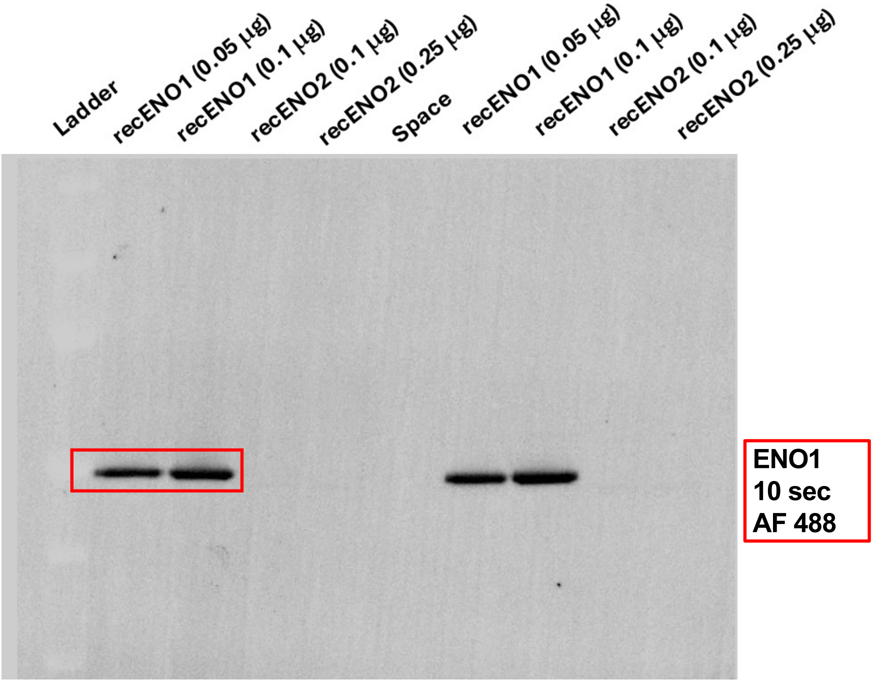

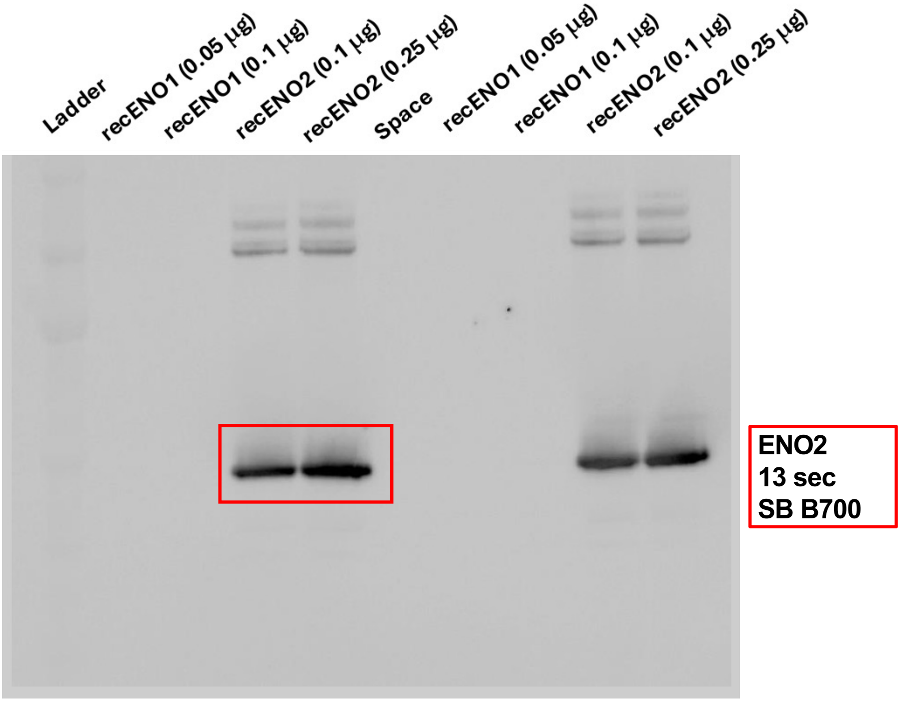

Recombinant ENO1 and ENO2, which have similar molecular weights, were probed on the same membrane using different specific primary antibodies and corresponding fluorescently labeled secondary antibodies. Wavelengths were detected in a BioRad ChemiDoc Imaging System using different filters.

Supplementary Figure S14: Original Western blot images for Figure 4A and 4B

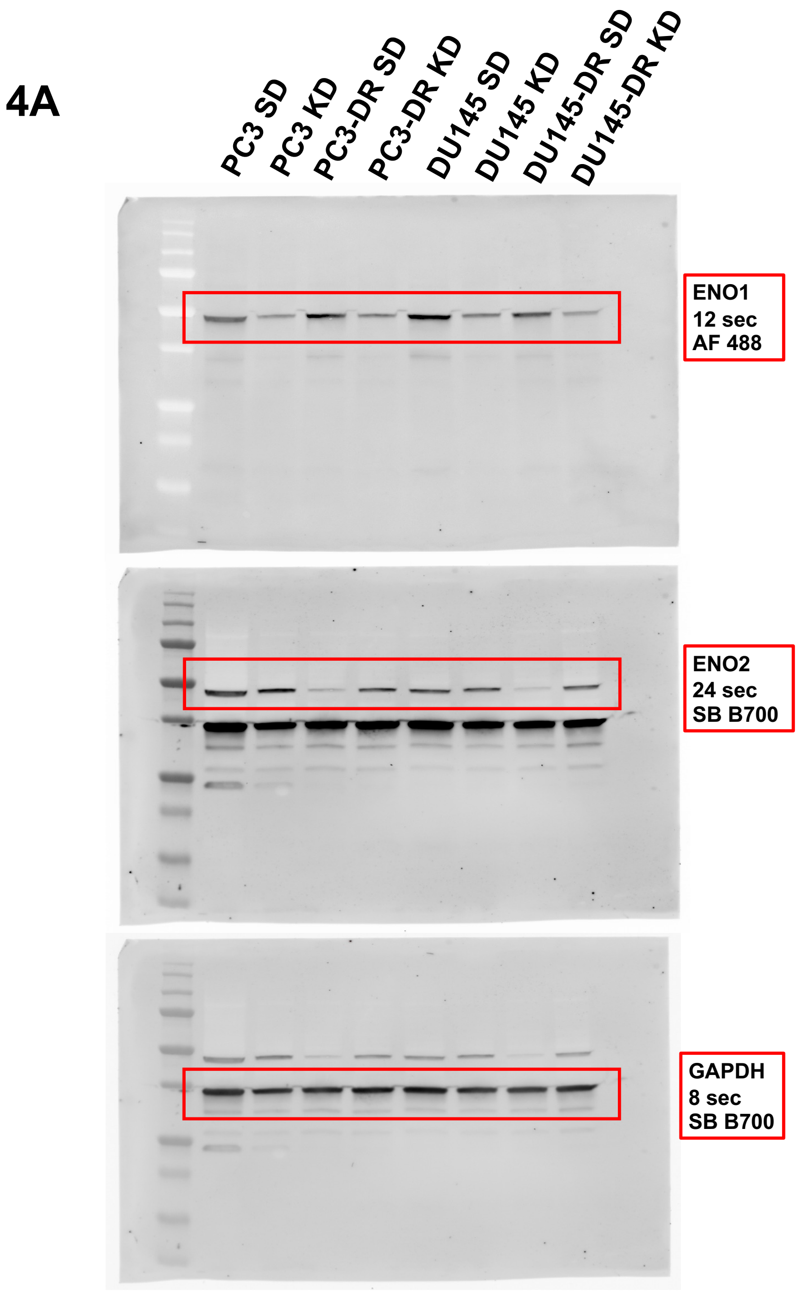

The three images are from the same membrane. ENO1 and ENO2, which have similar molecular weights, were probed on the same membrane using different specific primary antibodies and corresponding fluorescently labeled secondary antibodies. Wavelengths were detected in a BioRad ChemiDoc Imaging System using different filters.

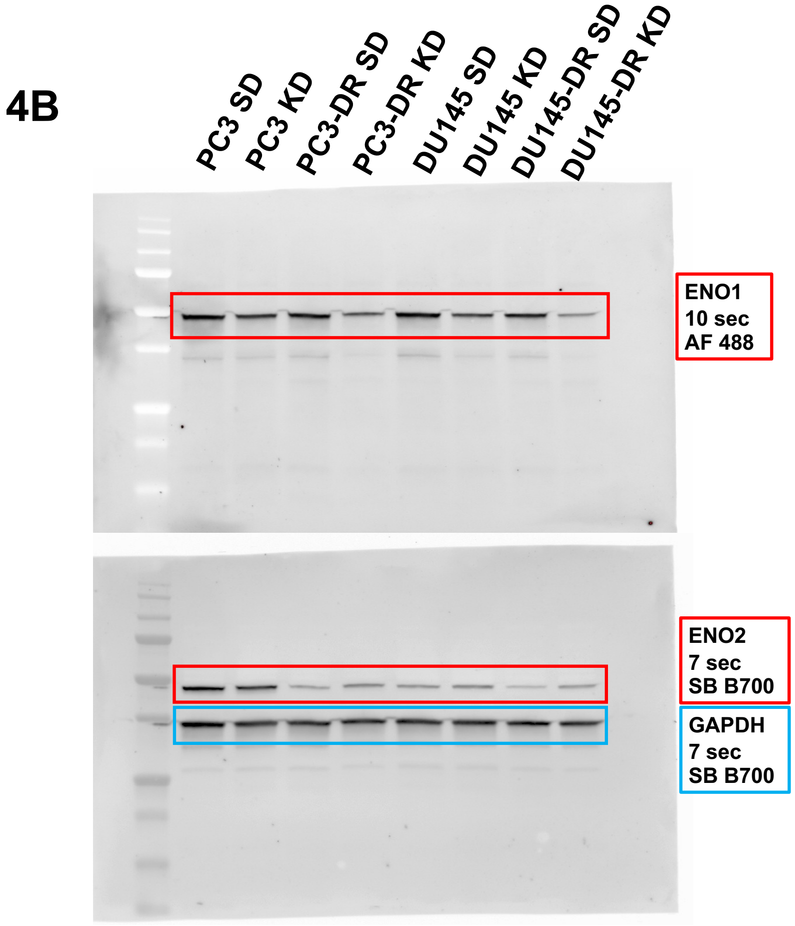

Both images are from same membrane using different specific primary antibodies and corresponding fluorescently labeled secondary antibodies. Wavelengths were detected in a BioRad ChemiDoc Imaging System using different filters.

Supplementary Figure S15: Original Western blot images for Figures 7C, D

7C

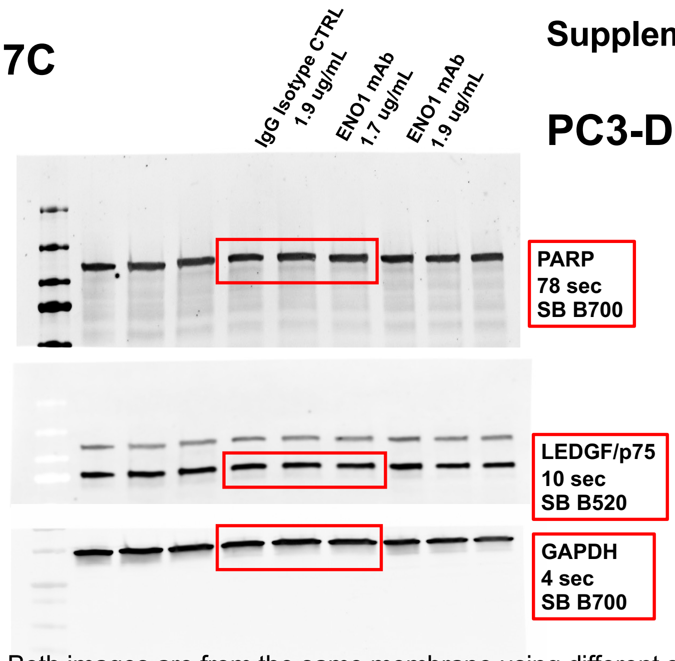

Both images are from the same membrane using different specific primary antibodies to PARP, LEDGF/p75, and GAPDH and corresponding fluorescently labeled secondary antibodies. Wavelengths were detected in a BioRad ChemiDoc Imaging System using different filters. The top band on the LEDGF/p75 blot corresponds to PARP.

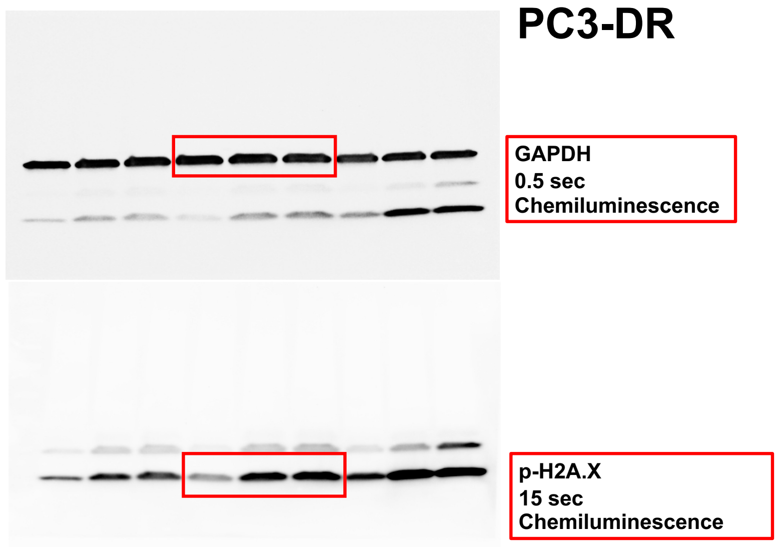

Both images are from the same membrane using different specific primary antibodies to GAPDH and p-H2A.X, with bands detected in a BioRad ChemiDoc Imaging System by chemiluminescence. The bottom band on the GAPDH blot corresponds to p-H2A.X.

7D

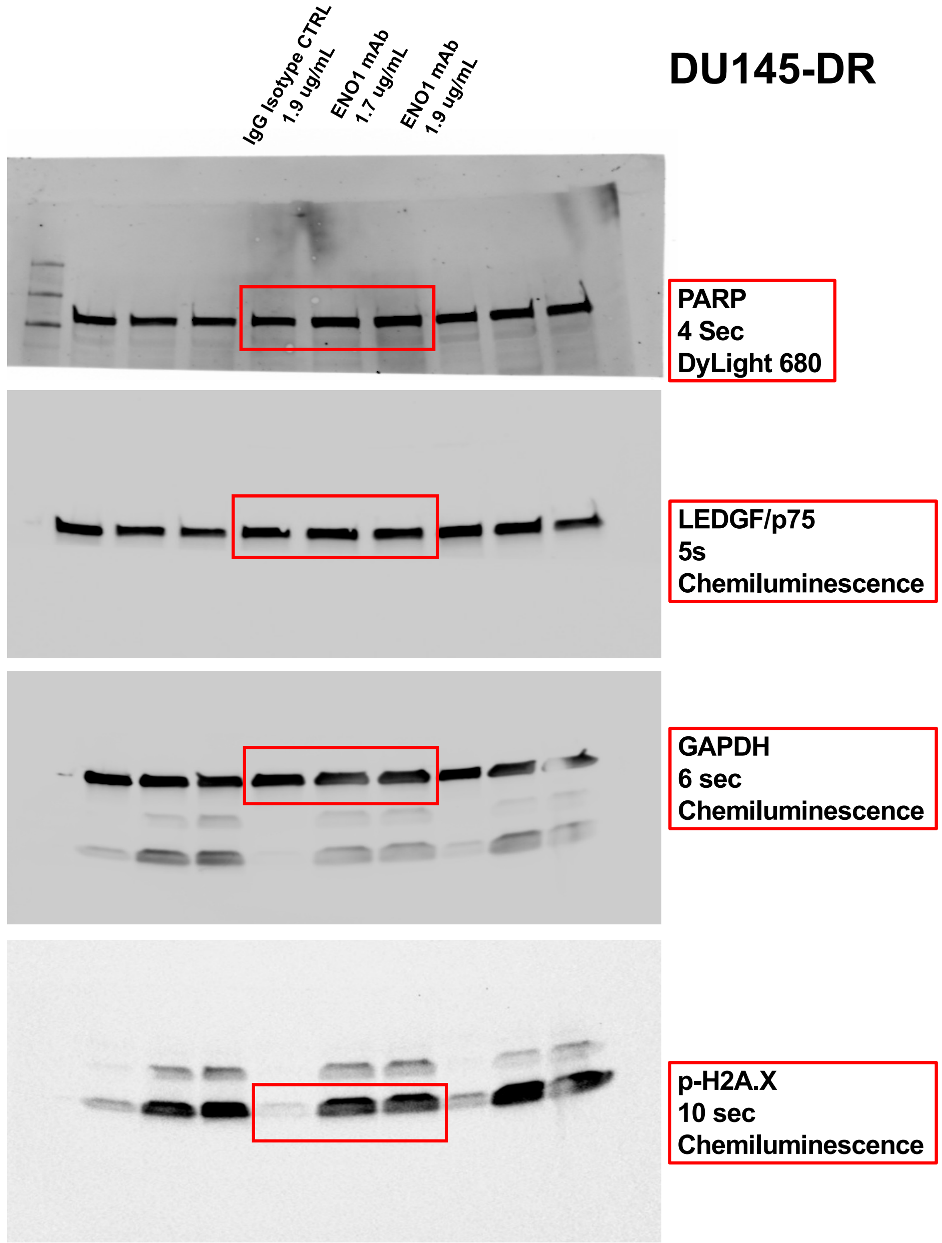

The four images are from the same membrane using different specific primary antibodies to PARP, LEDGF/p75, GAPDH and p-H2A.X. Bands were detected either by fluorescence (for PARP) or chemiluminescence in a BioRad ChemiDoc Imaging System using corresponding secondary antibodies. The bottom band on the GAPDH blot corresponds to p-H2A.X.

Supplementary Figure S16: Original Western blot images for Figures S1A and S1B

S1A

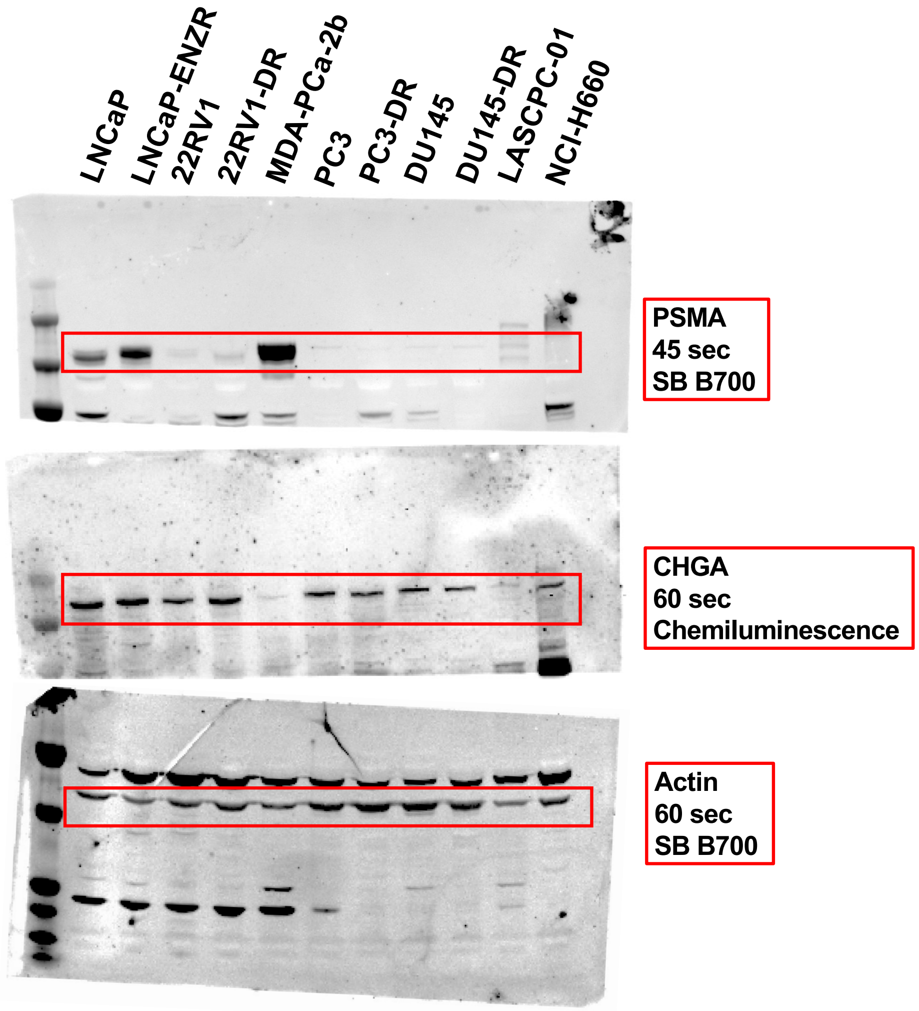

The three images are from the same membrane using different specific primary antibodies to PSMA, CHGA, and Actin. Bands were detected either by fluorescence (for PARP) or chemiluminescence in a BioRad ChemiDoc Imaging System using corresponding secondary antibodies. The bottom band on the GAPDH blot corresponds to p-H2A.X.

S1B

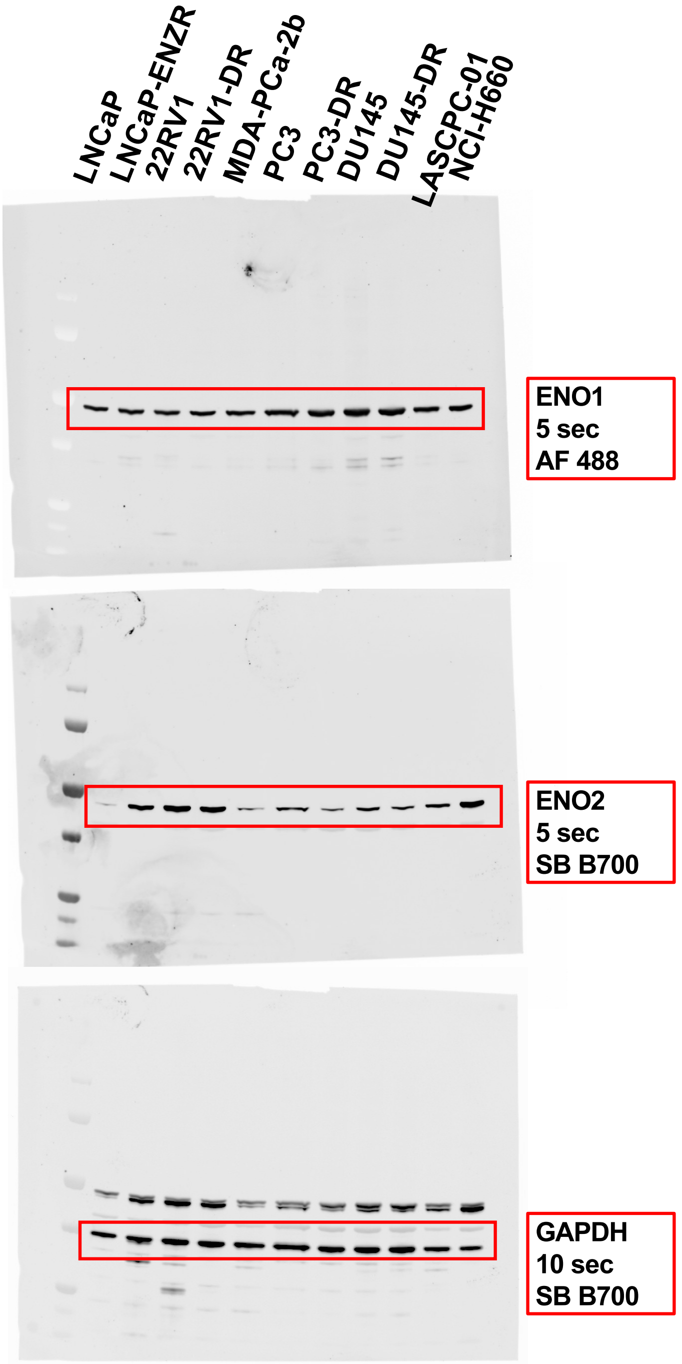

The three images are from the same membrane using different specific primary antibodies to ENO1, ENO2, and GAPDH. Bands were detected by fluorescence in a BioRad ChemiDoc Imaging System using the corresponding secondary antibodies.

Supplementary Figure S17: Original Western blot images for Supplementary Figure S1C

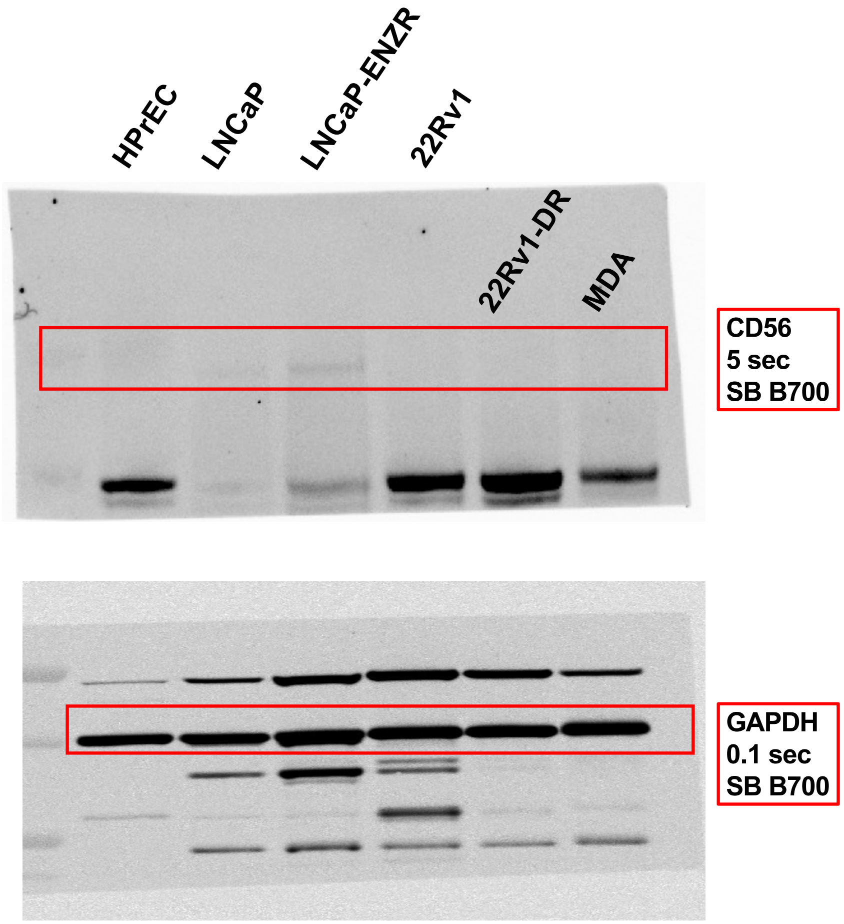

The two images are from the same membrane using different specific primary antibodies to CD56 and GAPDH. Bands were detected by fluorescence in a BioRad ChemiDoc Imaging System using the corresponding secondary antibodies.

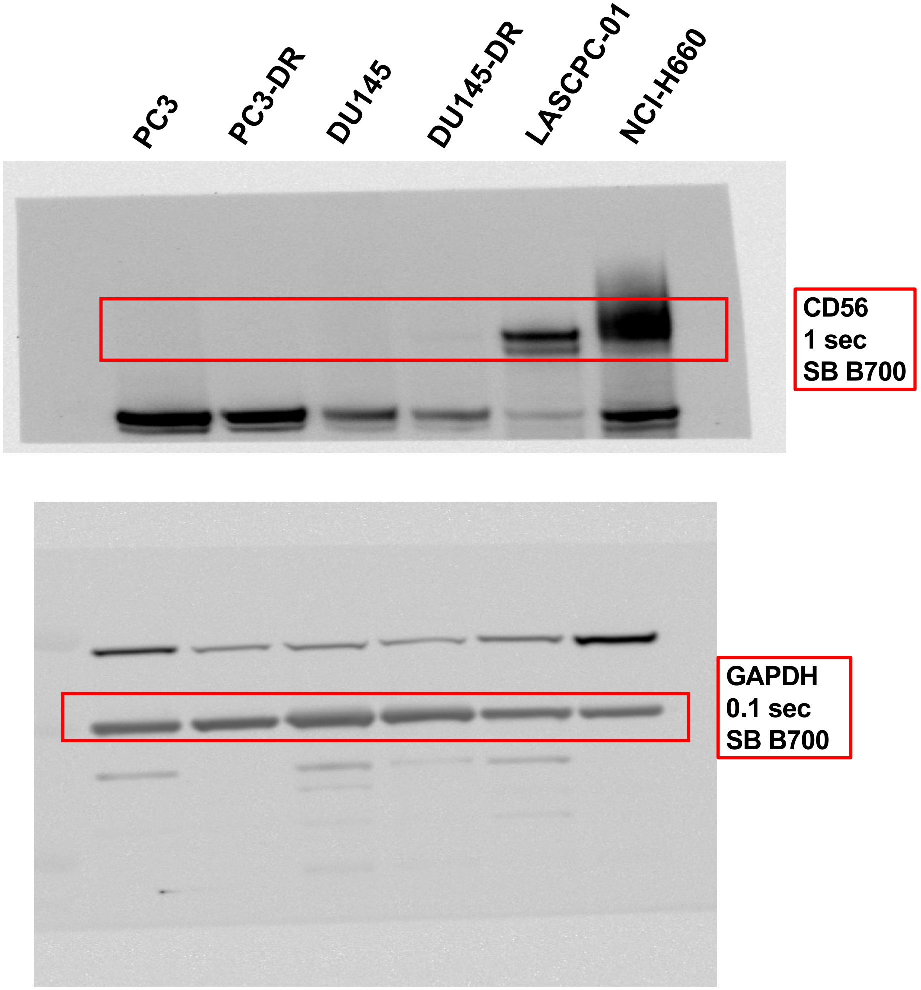

The two images are from the same membrane using different specific primary antibodies to CD56 and GAPDH. Bands were detected by fluorescence in a BioRad ChemiDoc Imaging System using the corresponding secondary antibodies.
